# Research progress on calixarene/pillararene-based controlled drug release systems

**DOI:** 10.3762/bjoc.21.139

**Published:** 2025-09-03

**Authors:** Liu-Huan Yi, Jian Qin, Si-Ran Lu, Liu-Pan Yang, Li-Li Wang, Huan Yao

**Affiliations:** 1 School of Pharmaceutical Science, Hengyang Medical School, University of South China, Hengyang 421001, China

**Keywords:** aromatic macrocycle, controlled-release drug delivery systems, stimulus response, supramolecular chemistry

## Abstract

Intelligent controlled-release drug delivery systems that are responsive to various external stimuli have garnered significant interest from researchers and have broad applications in the biomedical field. Aromatic macrocycles, including calixarenes and pillararenes, are considered ideal candidates for the construction of supramolecular drug delivery systems because of their simple synthesis, ease of modification, electron-rich and hydrophobic cavities, and highly selective molecular recognition. In recent years, numerous supramolecular drug delivery systems utilizing aromatic macrocycles have been developed. This review article provides an overview of the advancements of controlled drug release systems based on host–guest selective recognition, self-assembly, and nano-valves by the use of of calixarenes and pillararenes from five perspectives: pH, light, enzyme, hypoxia, and multi-stimuli combination responses. Furthermore, the article projects the future clinical application prospects of controlled-release technologies, with the aim of offering a reference for the utilization of aromatic macrocycles in drug-controlled release applications.

## Introduction

Drugs are defined by the Food and Drug Administration (FDA) as substances used for diagnosing, relieving, treating, or preventing diseases [1]. Traditional forms of drugs typically have a systemic effect, reaching both healthy and diseased areas, leading to a lack of selectivity, low bioavailability, and limited efficacy [2–4]. Nowadays, there are technologies that can better confine the action of drugs to where they are needed. Drug delivery is a technology that administers drugs to patients, which can specifically increase the concentration of drugs in certain parts of the body, thereby enhancing the therapeutic effect [5]. However, conventional drug delivery systems (such as capsules [6], tablets [7], ointments [8], etc.) have poor bioavailability, with fluctuating plasma drug levels and an inability to achieve sustained release. The emergence of controlled-release drug delivery systems has provided the possibility to improve patients' adaptability to medications [8–10]. By using biomedically compatible materials to carry drugs, these systems can release drugs at a controlled, uniform rate, maintaining stable blood drug concentrations, thereby fully exerting the therapeutic effects of the drugs [11–13]. The technology for modern controlled-release drug delivery systems began with the launch of Spansule sustained-release capsules in 1952. Since then, the development of various drug delivery systems has accelerated significantly over the past few decades. These systems include dissolution-controlled, diffusion-controlled, osmotic-controlled (which encompasses osmotic pressure and swelling control), chemically controlled, nanoparticle systems, and supramolecular controlled-release systems. Among these, supramolecular controlled-release systems have advantages such as dynamic adjustability, stimulus responsiveness, specific recognition and binding, and multi-target synergistic action [14–16]. These characteristics enable precise control of drug release and targeted drug delivery, thereby improving drug stability and bioavailability, and reducing drug toxicity and side effects.

Supramolecular chemistry, with molecular recognition at its core [17], utilizes non-covalent interactions such as electrostatic interactions, hydrogen bonding [18], and hydrophobic effects to provide valuable tools for developing new biomaterials and drug delivery systems [18–22]. This article categorizes supramolecular chemistry-based drug-controlled release systems into the following three mechanisms: (1) Drugs are identified and combined with supramolecular hosts through host–guest interactions [23–28]. By adjusting the conditions surrounding the guest molecules, such as changes in pH, light, and enzyme activity, the binding affinity between the guest and host molecules can be altered, thereby achieving controlled drug release and targeted delivery. (2) Drugs are loaded into self-assembled host–guest systems [29–32]. The chemical structure or properties of the host or guest molecules are altered upon exposure to specific stimuli, such as light, pH changes, or enzymes. This modification induces the disassembly of the host–guest complex, thereby releasing the encapsulated drugs. Fundamentally, this mechanism relies on controlling the assembly and disassembly processes. For example, supramolecular self-assembly technology enhances the targeting of chemotherapeutic drugs to tumor tissues, reducing systemic adverse reactions. (3) Macrocyclic aromatic supramolecular nano-valves have a pseudo-rotaxane structure with host–guest coordination and the kinetic properties of supramolecular interactions [33–34]. Different external stimuli, including pH changes, enzymes, light irradiation, hypoxia, and multi-stimuli responses, can alter the supramolecular structure or binding affinity to activate the opening and closing of the nano-valves. In addition to the three mechanisms that will be detailed in this article, researchers have made progress in the transport of amino acids and molecular peptides across membranes using macrocycles in recent years [35–37]. These works have paved the way for the potential application of remote-controlled membrane transport technology in the field of hydrophilic functional biomolecules, and is expected to be applied to stimulus-responsive controlled drug transport across membranes in the future. Currently, various artificially synthesized macrocyclic hosts have found extensive applications in drug-controlled release systems, for example, classic macrocycles like cyclodextrins [38] and cucurbiturils [39]. In recent years, aromatic macrocycles such as calixarenes (CAs) [40] and pillararenes (PAs) [41] have attracted widespread attention from the industry and scientists due to their simple synthesis which only require a one-step synthesis, convenient modification that can be achieved by introducing functional groups, electron rich and hydrophobic cavities that can effectively recognize electron deficient or neutral guest molecules, and high selectivity in binding with the guest. Their applications are extensive in the domain of drug-controlled release.

**Figure 1:**
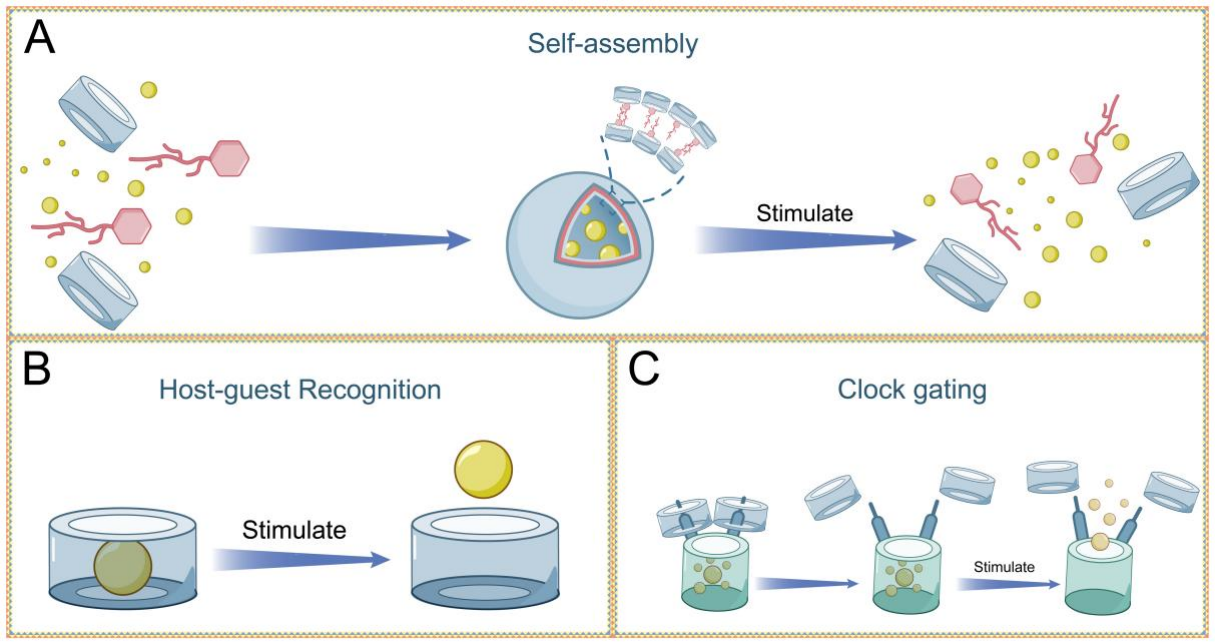
Schematic diagram of drug-controlled release mechanisms based on aromatic macrocycles.

This article mainly reviews the supramolecular drug delivery systems constructed from aromatic macrocycles (Figure 1). These systems achieve intelligent control over drug release through reasonable manipulation of stimuli, including external factors such as pH, light, enzymatic activity, hypoxia-triggered, and multi-responsive triggers. This article, based on the mechanisms of host–guest recognition, multifunctional assembly, and supramolecular nano-valves, provides several representative examples of CAs and PAs in the field of drug-controlled release. Finally, the article looks forward to future developments in this research area.

## Review

### 1 Aromatic macrocycles for host–guest drug release systems

Aromatic macrocycles are highly valuable in fields such as chemistry, materials science, and life science due to their rigid, electron-rich and hydrophobic cavities, and ease of modification [42]. Among these, CAs and PAs are representative aromatic macrocycles. They can be made water-soluble and exhibit good biocompatibility through carboxyl or phosphate group modification, showing great potential in biomedical carrier applications. Compared with other artificial macrocycles such as crown ethers [43], cyclodextrins, and cucurbiturils, CAs and PAs have unique advantages. Their synthesis is simple (one-step completion), and they have adjustable conformations and π-electron-rich cavities [44–45], enabling efficient recognition of electron-deficient or neutral guest molecules and high-selectivity binding in drug-controlled release. Many calix/pillar[*n*]arene hosts are soluble in water, particularly macrocycles that are modified with carboxyl and phosphate groups. Moreover, their amphiphilic modification ability allows easy self-assembly into functional materials like vesicles, micelles and nanoparticles, overcoming the difficulties in modifying cyclodextrin and cucurbituril. These features make CAs and PAs promising candidates for stimulus responsive drug release systems [46]. Their structural diversity and functional designability offer broad prospects for targeted applications. This section will focus on the structural features and applications of CAs and PAs.

#### 1.1 CAs: structure and properties

**Figure 2:**
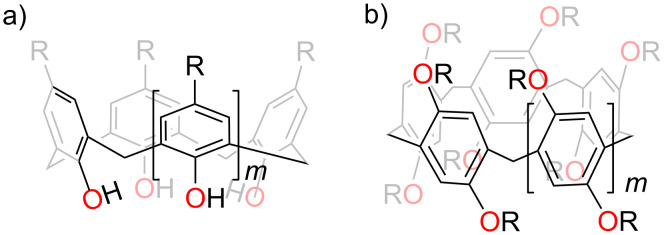
Chemical structure of a) calix[*n*]arene (*m* = 1,3,5), and b) pillar[*n*]arene (*m* = 1,2,3).

CAs are the third generation of supramolecular hosts [40], succeeding cyclodextrins and crown ethers. They are macrocycles composed of phenolic units condensed with formaldehyde, connected via methylene bridges. The most common CAs usually consist of 4, 6, or 8 phenolic units (Figure 2) [47–53]. The upper rim of CAs is equipped with hydrophobic alkyl groups, which, together with the benzene rings, form a hydrophobic cavity. In contrast, the lower rim consists of a series of neatly arranged hydrophilic phenolic hydroxy groups that can be selectively modified through chemical reactions [49].

CAs feature large hydrophobic cavities whose size can be precisely modulated by varying the number of aromatic rings. This structural tunability enables the cavities of CAs to selectively bind drugs of different sizes through hydrophobic and π–π stacking interactions and undergo targeted modifications to enhance their affinity for various guest molecules, such as choline [54], quaternary ammonium salts [55], drug molecules [56], etc. Although they generally have poor solubility, this can be improved through derivatization. Water-soluble calixarene derivatives can be obtained through functional modifications, including the introduction of sulfonic acid, amine, and carboxylic acid groups [57–59]. These water soluble macrocycle derivatives can be used to increase the solubility of drugs. The modified CAs encapsulate drug molecules within the host through host–guest interactions. The interactions between the drug and the calixarene enhance the solubility of the drug [60]. Further functionalization of these CAs' upper and lower rims with diverse moieties (e.g., amides, imines, sulfur-containing groups, or alkyl chains) significantly expands their multifunctionality and broadens their application scope. Notably, CAs can be readily modified into amphiphilic macrocycles that can self-assemble with guests, and it exhibits superior advantages over crown ethers and cyclodextrins in terms of controllable functionalization [61]. In clinical applications, particularly for anticancer drug conjugation, CAs demonstrate remarkable cancer cell selectivity, minimized off-target effects, enhanced delivery efficiency, and reduced systemic toxicity. Additionally, their synthetic accessibility, structural adaptability, and responsiveness to external stimuli (pH, light, enzyme, hypoxia) facilitate sustained drug release, side effect mitigation, and therapeutic efficacy, making them increasingly prominent in drug carrier research.

In recent years, the diversity of supramolecular amphiphiles has significantly expanded, bringing revolutionary breakthroughs to drug delivery systems. These structures, which possess both hydrophobic and hydrophilic characteristics, can self-assemble through non-covalent interactions to form well-defined aggregates such as micelles, vesicles, and nanoparticles [62]. Their morphologies mainly depend on molecular structure, concentration, and environmental properties. The cylindrical geometry of vesicles [63] promotes the arrangement of amphiphilic molecules with their hydrophobic tails pointing inward and hydrophilic heads pointing outward, forming a closed bilayer [64]. In contrast, micelles are usually assembled from single-tailed, conical molecules, where the smaller hydrophobic chains and larger head groups form monolayer spherical or rod-like structures [65]. Moreover, nanoparticles can be constructed through the physical or chemical cross-linking of polymers (such as PLGA and PEG), inorganic materials (such as gold and silica), or biomolecules (lipids and proteins), enabling efficient drug loading and controlled release [66–68]. Among them, amphiphilic CAs that incorporate both hydrophilic groups and hydrophobic alkyl chains through dual modifications [69]. These derivatives have emerged as a research hotspot due to their unique combination of water solubility, biocompatibility, and host–guest encapsulation capability [47,52,70]. The aggregation behavior of amphiphilic CAs is closely related to the size of the macrocycle, the length of the alkyl chain, and the nature of the polar head group [71]. Compared with traditional surfactants, amphiphilic CAs have a lower critical micelle concentration and are more likely to self-assemble into various forms of aggregates, such as spherical micelles [72], vesicles, and spherical nanoparticles [73]. Calixarene-based amphiphiles are characterized by their low cytotoxicity and ability to load drugs. The structure of calixarene-drug complexes can respond to external stimuli, causing the self-assembled complex to disassemble, which enables sustained drug release. Based on these characteristics, CAs hold potential for applications in drug delivery systems.

#### 1.2 PAs: structure and properties

Since Ogoshi et al. first introduced pillar[*n*]arenes in 2008, these macrocycles have become essential to the synthetic macrocyclic receptor field [74]. Research on PAs and their derivatives has become a hot topic [75]. PAs can be prepared via the Friedel–Crafts condensation, which involves combining 1,4-dimethoxybenzene with paraformaldehyde in the presence of a Lewis acid. The central axis of these aromatic hydrocarbon molecules possesses *n* fold rotational symmetry [76]. PAs possess highly modifiable ring positions, and many PA derivatives with different functional groups can be obtained through the cyclization reaction of 1,4-dialkoxybenzene monomers or post-synthetic modification reactions [74]. PAs possess a unique internal cavity characterized by an electron-rich and hydrophobic environment, which significantly enhances the density of π-electrons. This structural feature makes pillar[*n*]arenes particularly effective at encapsulating guest molecules that are either electron-deficient or neutral. For example, pillar[5]arene (PA5) has a strong binding affinity for neutral guest molecules in organic solvents, [77] which is not feasible for crown ethers, CAs, and resorcinarenes. In addition, PAs can form supramolecular systems in the following ways: (1) the electron-rich cavity interacts electrostatically with cationic guests, such as methyl viologen derivatives, pyridinium salts, and quaternary ammonium salts [78]. (2) After modification with cationic groups, they can bind with anionic molecules, such as sulfonates [79]. It is worth mentioning that the cavity of PAs can encapsulate photo-responsive guests such as cationic azobenzene derivatives, providing new ideas for the innovation of drug design and therapeutic strategies. Notably, PA derivatives also exhibit specificity in recognizing important molecules in biological processes, such as acetylcholine [80], amino acids [81], doxorubicin (DOX) [82] and antibiotics [83], etc. Among the many derivatives, water-soluble pillar[5]arene (WP5) and pillar[6]arene (WP6) have become representative systems for biomedical applications due to their excellent performance in drug-controlled release [84–85].

Recent research on drug delivery has focused more on amphiphilic PAs, which are mainly constructed through two strategies: one is self-assembly after separately modifying hydrophobic and hydrophilic segments; the other is the formation of host–guest complexes between water-soluble PAs and guest molecules containing hydrophobic chains, followed by self-assembly into bilayer vesicles through host–guest interactions, hydrophobic effects, and π–π stacking interactions [86]. Their amphiphilic nature enables these systems to enter cells efficiently through endocytosis, providing a new drug delivery approach and holding great potential in precision medicine due to their programmable and stimulus-responsive features.

Additionally, PAs can be hybridized with various inorganic materials, such as metal-organic frameworks (MOFs), mesoporous silica nanoparticles (MSNs), metal nanoparticles, and carbon materials [87]. By designing through electrostatic and non-covalent interactions, PAs can be used as "molecular switches" to construct mechanized MOFs with the framework of MOFs, realizing the controlled release of drugs [88]. When integrated with MSNs, nano valves consist of a mobile macrocyclic host that moves along a linear molecule between two or more binding sites. This movable macrocycle acts as a gate, controlling the movement of cargo into and out of the MSN pores [89]. These materials, with their ideal host–guest recognition characteristics, have provided new ideas for the design and synthesis of nano valve systems. These composite materials not only have the advantages of high specific surface area and large pore volume for drug loading, but also their unique stimulus responsive characteristics break through the limitations of traditional delivery systems: The pH, Zn^2+^, and competitive agent-responsive characteristics can disrupt the multi-responsive supramolecular nanovalve systems based on PA5, primarily by disrupting the high-affinity host–guest interactions between PA5 and these groups (mainly through phosphonate-quaternary ammonium ion pairing) [90]. Azobenzene guest molecules can achieve dual functions (on and off) as ligands through photo-induced cis-trans isomerization. Using pillar[6]arene as the ion channel, the host–guest complexation realizes a reversible ON-OFF-ON type pseudo-monomeric ligand-gated ion transport switch [91]. The development of these pillararene-based materials has enriched the dynamic regulation methods of supramolecular structures and provided innovative approaches for prodrug design.

### 2 Stimulus-responsive controlled release in supramolecular systems

Many efforts have been made to prepare stimulus-responsive drug carriers, including some aimed at disease-related stimuli [92]. In the field of supramolecular materials, various stimulus strategies have been introduced to regulate the construction and destruction of aromatic macrocycles and their components. The external triggers include: pH [93], oxidation–reduction states [94], enzymatic actions [95], and light [96]. Specifically, within host–guest frameworks, the capability to meticulously regulate the assembly of complexes and synchronize them with biologically pertinent or biocompatible stimuli holds substantial potential for augmenting the precision of therapeutic interventions [97–98]. In certain scenarios, these stimuli precipitate reversible alterations in the formation of host–guest complexes, while in other cases, they may catalyze irreversible disintegration to avert reversibility. Given the benefits of the chemical stability of most aromatic macrocycles, creating stimulus-responsive complexes usually requires the guest components to undergo stimulus-triggered changes that affect their ability to bind with the host macrocycles. The controllable release of drugs is one of the most important applications of stimulus-responsive supramolecular systems and contributes to targeted therapy. Drugs are loaded into supramolecular nanosystems formed by self-assembled host–guest systems. Under specific stimuli (light, enzymes, hypoxia, pH values, etc.), changes in the host or guest molecules' chemical structure or properties lead to the host–guest system's disassembly, thereby releasing the drugs from the supramolecular systems [99]. Thus, the core principle of controlled release lies in modulating the association and dissociation of host–guest interactions. Aromat-containing macrocycles, such as CAs and PAs, are deemed exceptional hosts for fabricating host–guest nanosystems due to their versatile characteristics and capacity to self-assemble into supramolecular architectures via non-covalent interactions. These host–guest nanosystems play a vital role in controlled release, nanotechnology, materials chemistry, artificial molecular machines, and synthetic ion channels. Numerous host–guest systems have been developed based on their responsiveness to particular external triggers, including pH, enzymes, hypoxia, and light. The following section will review and discuss some of the stimulus responses.

#### 2.1 pH-responsive controlled release

Changes in acidity are a typical stimulus for adjusting host–guest supramolecular nanosystems. It is widely recognized that the acidity levels of various organs, tissues, and cellular compartments differ. For example, the acidity of tumor and inflamed tissues is distinct from that of healthy tissues, offering a potential physiological trigger for pH-responsive drug delivery. Thus, drug release can be regulated by leveraging the variations in acidity between normal and diseased cells [100]. Developing therapeutic materials that react to shifts in pH, particularly the acidification arising from the endocytic pathway of internalized carriers or disease-induced acidification in local tissue microenvironments, is a prevalent approach for fabricating stimulus-sensitive drug carriers. Consequently, the construction of pH-responsive systems based on aromatic macrocycles has been widely promoted, with typical water-soluble carboxylated PAs and CAs frequently employed as host molecules or structural components [100]. This section will examine cases of pH-dependent degradation and drug release mechanisms.

**Figure 3:**
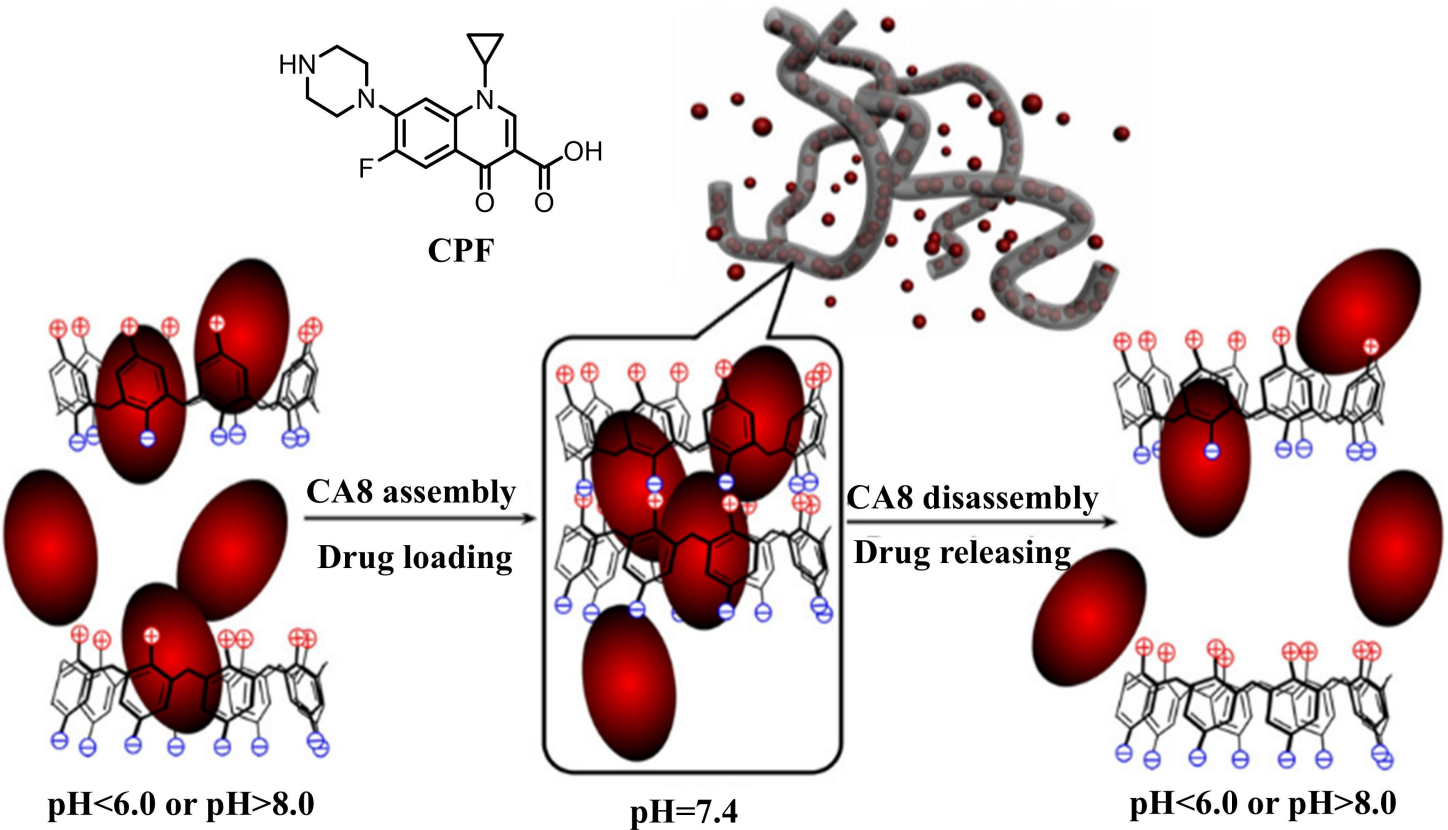
Changes in pH conditions cause the release of drugs from CA8 host–guest complexes [101]. Figure 3 was adapted with permission from [101], *Colloids and Surfaces B: Biointerfaces*, vol. 101, by Y. Xue; Y. Guan; A. Zheng; H. Xiao, “Amphoteric calix[8]arene-based complex for pH-triggered drug delivery”, pages 55–60, Copyright (2012), with permission from Elsevier. This content is not subject to CC BY 4.0.

Amphoteric CAs, known for their effective hydrophobic drug-loading capabilities, offer an excellent platform for controlled drug release. Researchers have synthesized amphoteric CAs to introduce pH-responsive properties, allowing each macrocycle to possess positively and negatively charged gates. Amphoteric CA8 were prepared by introducing positively charged groups at the lower rim of sulfonated CA8. The cavity size of the water-soluble CA8 matches with the hydrophobic drug ciprofloxacin (CPF). The efficient encapsulation of CPF (with a loading capacity of 17.8–24.5%) is achieved through the synergistic action of hydrophobic interactions and hydrogen bonding. Under physiological pH conditions (7.0–7.6), the CA8-CPF complex self-assembles into a stable multilayer structure through electrostatic interactions. When the environmental pH deviates from the neutral range (pH < 6.0 or pH > 8.0), the charge balance is disrupted, leading to the dissociation of the complex and the release of the drug. This pH-sensitive "charge switch" mechanism enables CA8 to precisely control the release behavior of CPF, providing new insights for the development of environmentally responsive antibiotic delivery systems (Figure 3) [101]. This pH-sensitive "charge switch" mechanism enables CA8 to precisely control the release behavior of CPF, providing new insights for the development of environmentally responsive antibiotic delivery systems.

**Figure 4:**
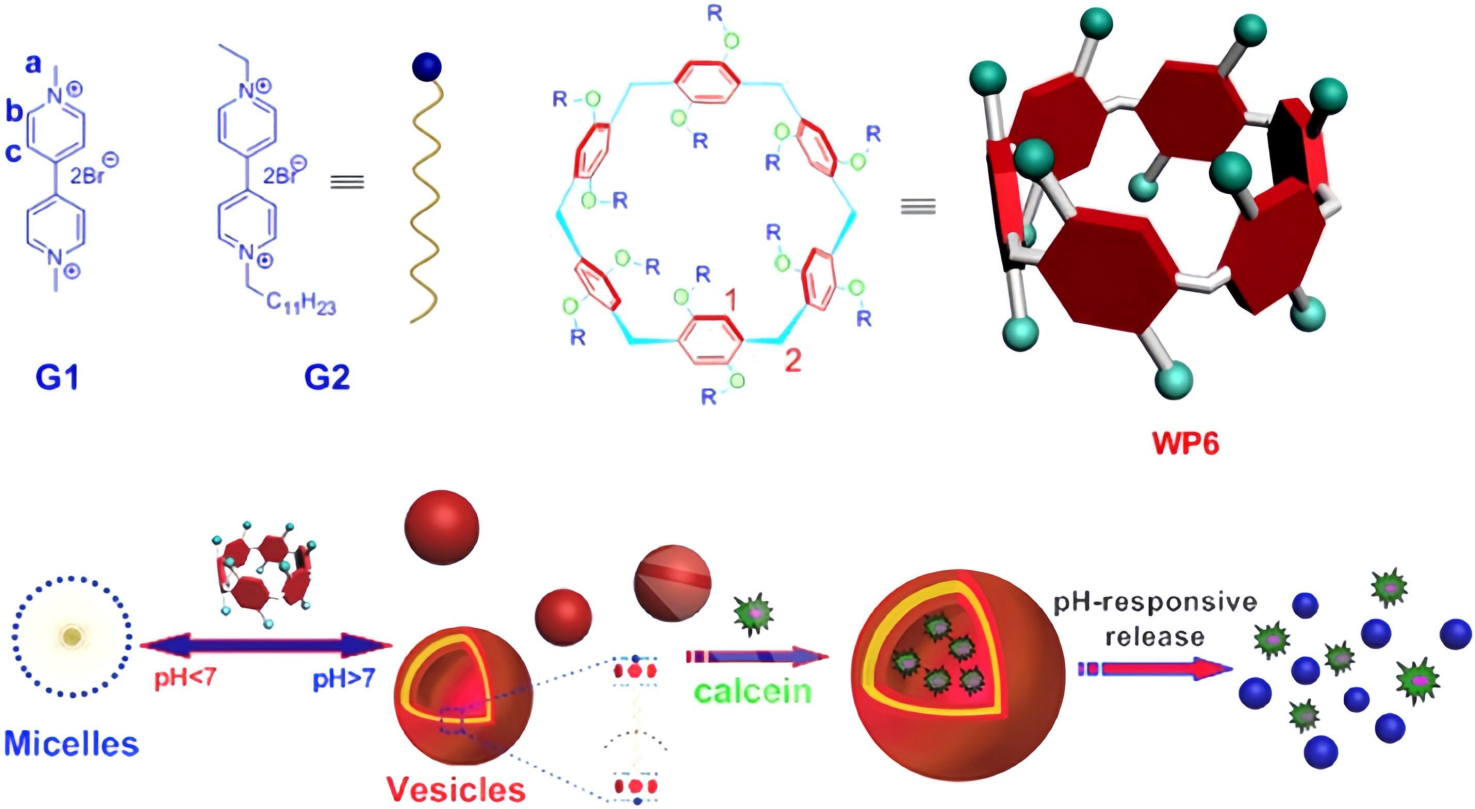
The illustration of the pH-mediated 1:1 complex formation between the host and guest molecules in aqueous solution, along with the pH-triggered release mechanism [78]. Figure 4 was adapted with permission from [78], Copyright 2012 American Chemical Society. This content is not subject to CC BY 4.0.

Water-soluble aromatic macrocycles are commonly utilized in drug delivery systems. Vesicles are valuable tools in biology, chemistry, and materials science. Supramolecular pH stimuli-responsiveness has applications at the cellular level and has the potential for controlling drug release in environmental protection. In 2012, Huang and colleagues [78] described the construction of supramolecular vesicles using WP6 and pyridine derivatives (G1) (Figure 4). This supramolecular system achieves controllable assembly and disassembly through pH responsiveness: under acidic conditions (pH 6.0), protonation of the WP6 carboxyl groups leads to vesicle disassembly; whereas in a neutral environment (pH 7.4), deprotonated WP6 can rebind with the guest molecules. The molecular recognition mechanism of this system is based on the synergistic effect of multiple non-covalent interactions, including electrostatic attraction, hydrophobic interactions, and π–π stacking. Researchers have further utilized this feature to achieve reversible transformation of amphiphilic guest molecules between micellar and vesicular states and have successfully applied it to the controlled release of dye molecules. Notably, the system can also effectively neutralize the toxicity of paraquat through host–guest interactions, providing a new approach for the treatment of environmental pollutants. This pH-responsive supramolecular platform shows broad application prospects in targeted drug delivery and environmental remediation.

Later, in 2014, Huang and colleagues synthesized a water-soluble pillar[7]arene (WP7) by adding 14 anionic carboxylic acid groups to both sides [102]. They explored the pH-dependent complexation of WP7 with paraquat derivative G1 in water. The host WP7 and guest G1 form a 1:1 pseudorotaxane, with an association constant of (2.96 ± 0.31) × 10^5^ M^−1^. Furthermore, they leveraged this new molecular recognition motif to create a supramolecular amphiphilic reagent using WP7 and an amphiphilic paraquat derivative G2. This pH-responsive drug delivery system has considerable potential for future medical applications. Hydrophilic supramolecular polymers that respond to stimuli, especially those formed in aqueous solutions, are potential candidates for mimicking biocompatibility or creating important functional materials.

**Figure 5:**
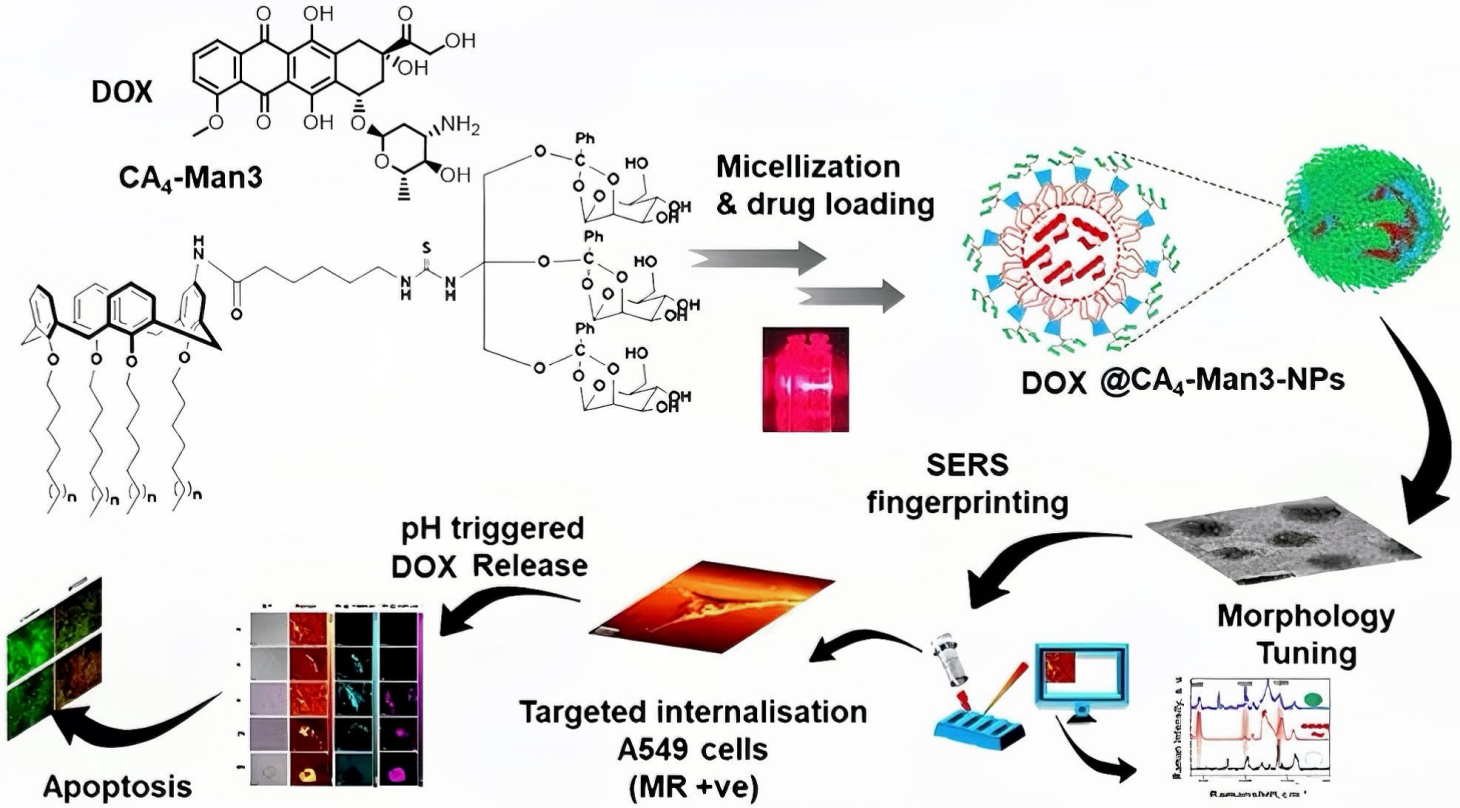
Illustration of the pH-responsive self-assembly of mannose-modified CA4 into micelles and the subsequent release of DOX [103]. Figure 5 was adapted with permission from [103], *Journal of Controlled Release*, vol. 339, by Sreedevi, P.; Nair, J. B.; Joseph, M. M.; Murali, V. P.; Suresh, C. H.; Varma, R. L.; Maiti, K. K, “Dynamic self-assembly of mannosylated-calix [4] arene into micelles for the delivery of hydrophobic drugs”, pages 284–296, Copyright (2021), with permission from Elsevier. This content is not subject to CC BY 4.0.

In 2021, Maiti et al. employed small-molecule mannose-modified CA4 to create the core structure CA4-Man3 and produce nano-micelles known as CA4-Man3-NPs (Figure 5) [103]. CA4-Man3-NPs achieve efficient targeted drug delivery through a unique self-assembly mechanism. This system leverages the unique polymeric characteristics of CA4, which reduces the critical aggregation concentration and enhance intermolecular orderliness to form structurally stable nanocarriers [52]. The key design features are: 1) The mannose groups at the upper rim confer specific recognition ability for cancer cell surface receptors; 2) The hydrophobic core formed by the alkyl chains at the lower rim and the hydrogen-bonding network constructed by the thiourea unit can efficiently encapsulate DOX. This structure integrates multiple non-covalent interactions, such as hydrophobic interactions and π–π stacking, endowing the micelles with both structural stability and pH responsiveness. When targeted to the tumor microenvironment (acidic pH), the core of the micelles dissociates to achieve specific release of DOX, significantly improving the delivery efficiency and tumor targeting of hydrophobic drugs.

**Figure 6:**
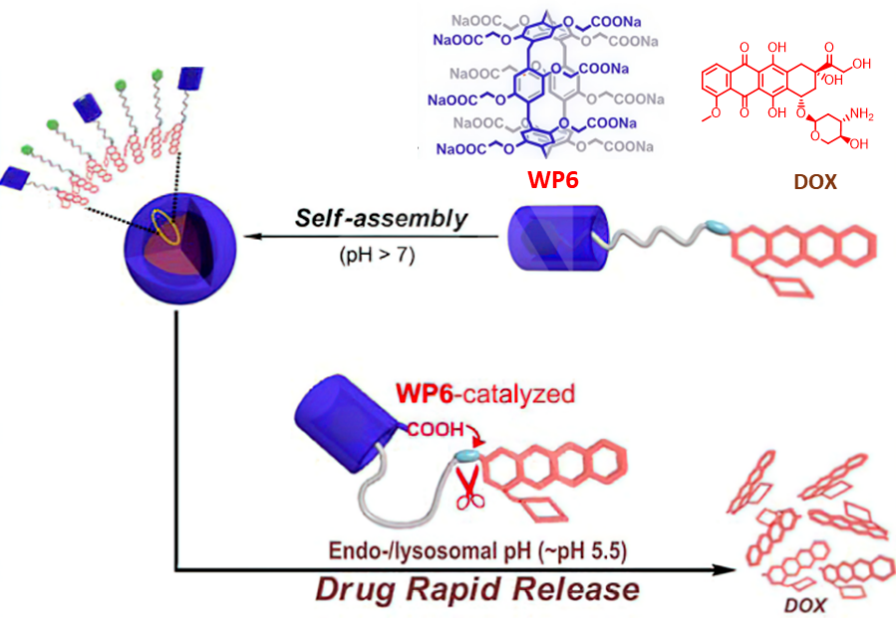
Illustration of the assembly of supramolecular prodrug nanoparticles from WP6 and DOX-derived prodrugs (G3 and G4) for WP6-mediated rapid release of the drug [104]. Figure 6 was adapted with permission from [104], Copyright 2015 American Chemical Society. This content is not subject to CC BY 4.0.

The release of DOX from PAs also can be regulated by a pH-responsive mechanism. In 2015, Wang and coworkers synthesized a novel supramolecular prodrug nanoparticle exploiting the host–guest interactions between WP6 and DOX-derived prodrugs (Figure 6) [104]. As demonstrated in previous studies, WP6 has shown good biocompatibility and acid-responsive properties in aqueous media [78,105]. Meanwhile, it has been proven that WP6 has a strong binding [106–107] affinity for pyridinium salts in water driven by hydrophobic and electrostatic interactions. DOX-based prodrugs are synthesized by directly conjugating hydrophobic DOX with pyridinium-modified flexible alkyl chains (G3) or short EGn (ethylene glycol) chains (G4) via acid-cleavable hydrazone bonds. The above-mentioned WP6-G3 and WP6-G4 supramolecular complexes, based on their amphiphilic nature, have the ability to form higher-order aggregates in weakly alkaline phosphate-buffered saline (PBS). The resulting supramolecular nanoparticles exhibited stability under physiological conditions. The cumulative release of DOX reached nearly 100% within 30 minutes at a pH of 5.5, which simulates the lysosomal environment at 37 °C. Both CAs and PAs are effective supramolecular platforms for the controlled release of DOX, demonstrating significant potential for various applications.

**Figure 7:**
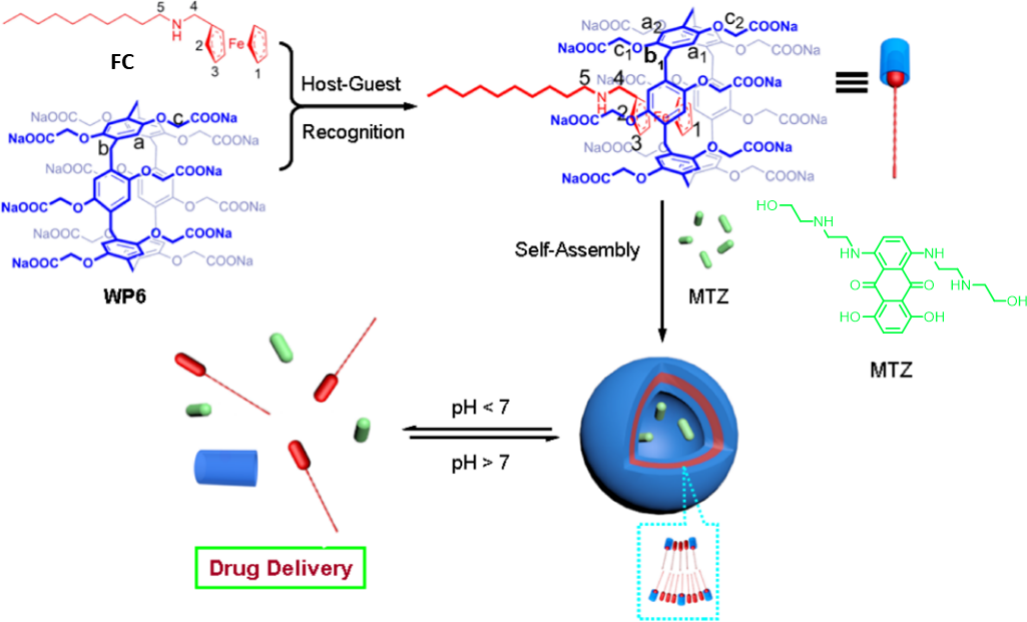
Illustration of the formation of supramolecular vesicles and their pH-dependent drug release [93]. Figure 7 was adapted with permission from [93], Copyright 2013 American Chemical Society. This content is not subject to CC BY 4.0.

In 2013, Wang and others reported [93] that WP6 and hydrophobic ferrocene derivatives (FC) self-assembled into supramolecular vesicles loaded with mitoxantrone (MTZ) exhibiting significant pH-responsive behavior in aqueous solutions, exceptionally rapid release of MTZ in a low pH environment (Figure 7). The complex exhibits significant amphiphilicity: the carboxylate residues of WP6 confer hydrophilicity, while the alkyl chains of FC contribute hydrophobicity, driving the molecules to self-assemble into supramolecular vesicles in the aqueous phase. The vesicle structure features a bilayer characteristic, with two hydrophilic carboxylate shell layers enveloping a hydrophobic alkyl chain core. The assembly driving force directly relies on the host–guest interaction between WP6 and FC. Further studies have shown that by regulating the deprotonation/protonation state of the WP6 carboxyl groups through pH control, the reversible dissociation and reassembly of the vesicle structure can be achieved, thereby constructing a pH-responsive drug carrier. The novel supramolecular vesicles assembled from WP6 and guest molecules in aqueous environments are promising for controlled release and drug delivery applications. They have established a foundation for pH-responsive controlled release systems based on WP6, encouraging increased research participation within this domain.

**Figure 8:**
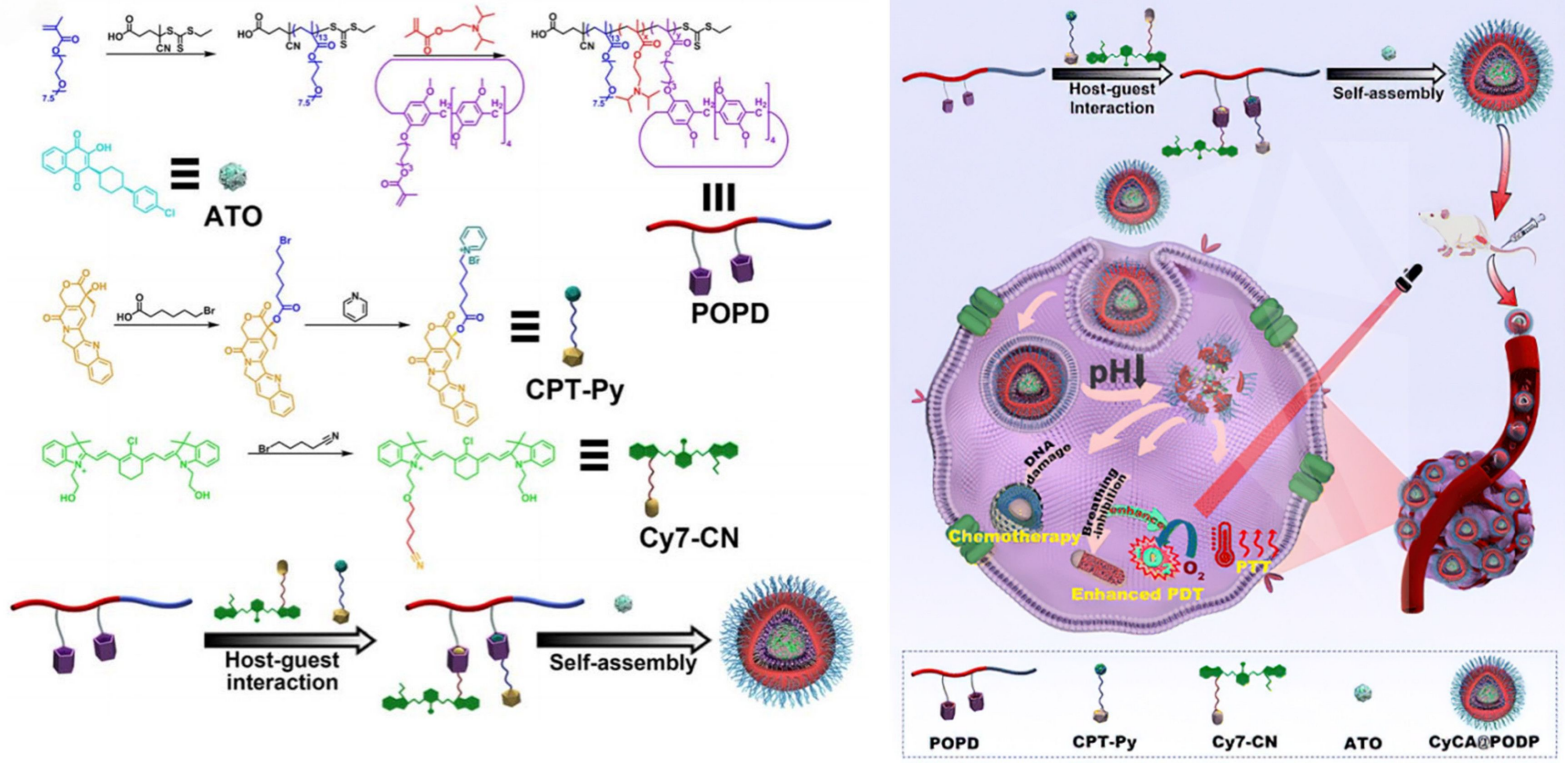
Schematic illustration of the application of the multifunctional nanoplatform CyCA@POPD in combined PDT, photothermal therapy (PTT), and chemotherapy [109]. Figure 8 was reproduced from [109] (“A supramolecular nanoplatform for imaging-guided phototherapies via hypoxia tumour microenvironment remodeling”, © 2023 W. J. Zhou et al., published by The Royal Society of Chemistry, distributed under the terms of the Creative Commons Attribution-NonCommercial 3.0 Unported License, https://creativecommons.org/licenses/by-nc/3.0/). This content is not subject to CC BY 4.0.

Photothermal construction and manipulation of optical materials are crucial in pharmaceutical or biological fields. In 2016, Zhang and colleagues [108] developed a supramolecular nanovesicle with improved photodynamic therapy (PDT) capabilities. This system was based on the host–guest interactions between polyethylene glycol-functionalized pillar[5]arene (PEG-P[5]A) and a pyridinium-capped porphyrin derivative containing a disulfide bond (TPPC6-SS-Py). The supramolecular amphiphile could self-assemble into spherical micelles, demonstrating excellent colloidal stability in aqueous solutions, as evidenced by transmission electron microscopy (TEM) and dynamic light scattering (DLS). The PEG-P[5]A/TPPC6-SS-Py micelles showed rapid release of the porphyrin photosensitizer under reducing conditions. Furthermore, in 2023, Zhang and colleagues [109] constructed a tumor microenvironment (TME)-activated supramolecular nanoplatform (Figure 8) which was consisted of a pillar[5]arene-based amphiphilic polymer (POPD), a phototherapeutic agent (Cy7-CN), an antimalarial drug with respiratory function (atovaquone, ATO), and a chemotherapeutic agent (pyridinium camptothecin, CPT-Py). This platform was designed for imaging-guided phototherapy to alleviate hypoxia. They created a new supramolecular amphiphile that enabled rapid drug release and efficient cellular internalization, potentially providing a robust platform for drug delivery and controlled release systems.

The excellent biocompatibility of both supramolecular vesicles and nano-fragments with the body indicates that aromatic macrocycles have great potential for biomedical applications. These macrocycles are distinguished by their unique structures and superior performance in host–guest interactions. Creating functional and intelligent supramolecular nano-drug carriers for controlled drug delivery is promising. In this context, water-soluble carboxylated arenes are the primary host molecules for most pH-responsive nanosystems. The nanosystem is broken down by the highly acidic environment of tumor cells, which acts as an exogenous or endogenous stimulus to enable the controlled release of the encapsulated drugs. These nanosystems are highly compatible with the body and comprise biocompatible, water-dispersible, and low-toxicity small molecules.

#### 2.2 Light-responsive controlled release

Light is an ideal external stimulus due to its ease of use, low cost, and safety [110–111]. Its unique advantages include being easily turned on and off, providing high spatial and temporal resolution with precise control over irradiation wavelength and intensity, and facilitating rapid responses in confined spaces [111]. Its tunable parameters, such as wavelength, duration, and intensity, along with these characteristics, have contributed to its extensive application in innovative materials and molecular devices. Ultraviolet light, in particular, is commonly employed as an external trigger for supramolecular systems. Azobenzene can regulate geometry, shape, and interfacial curvature under ultraviolet or visible light irradiation. It undergoes *cis*–*trans* isomerization, which can trigger alterations in bioactivity or assembly configurations. Methods for creating photo-responsive cages or capsules include fabricating cages and capsules from photoactive building blocks, and developing cages and capsules encapsulating photo-responsive guest molecules [112–113]. Incorporating supramolecular amphiphiles into photo-sensitized systems has propelled the creation of novel multifunctional photo-responsive materials. This section will discuss drug molecule capture and release systems based on two hosts.

**Figure 9:**
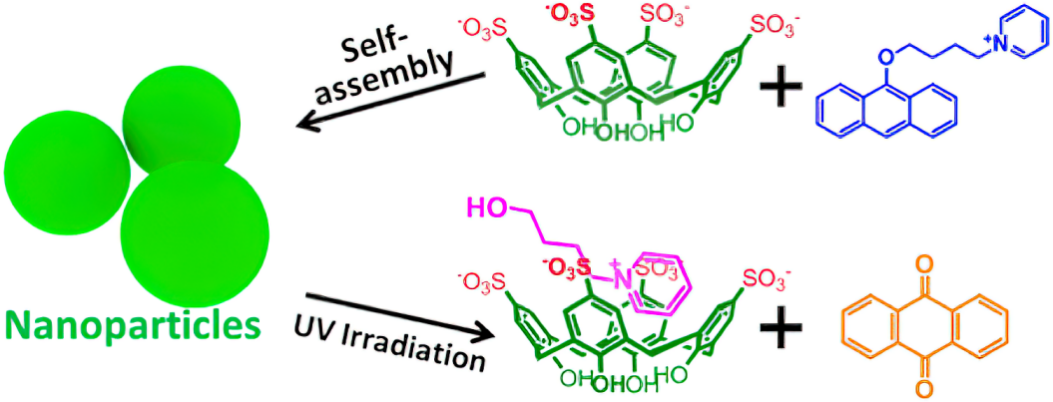
Illustration of the photolysis of an amphiphilic assembly via CA-induced aggregation [114]. Figure 9 was reprinted with permission from [114], Copyright 2015 American Chemical Society. This content is not subject to CC BY 4.0.

The photo-sensitive assembly has drawn considerable interest in degradable materials, drug delivery systems, and tissue engineering. Anthracene (AnPy) derivatives are recognized for their high reactivity when exposed to light and are frequently employed as photo-degradable molecules. Liu described a photo-sensitized system composed of amphiphilic 9-alkoxy-substituted AnPy and *p*-sulfonato calix[4]arenes (SC4A) (Figure 9) [114]. SCnAs have a unique propensity to regulate the aggregation behavior of aromatic or amphiphilic molecules by reducing the critical aggregation concentration, enhancing the compactness of aggregates, and modulating the orderliness within aggregates, which is referred to as calixarene-induced aggregation (CIA) [52]. Taking the SC4A-AnPy system as an example, SC4A encapsulates the pyridine group of AnPy with its electron-rich cavity, neutralizing the electrostatic repulsion of the cationic head group and promoting the close packing of the AnPy rings through hydrophobic interactions and π-π stacking, while its deprotonated phenolic groups maintain water solubility. This assembly approach not only suppresses the fluorescence self-quenching of AnPy in the aggregated state (enhancing the yield of singlet oxygen) [115], but also boosts its photo-sensitivity – under light irradiation, the anthracene in the complex can be efficiently converted to the active drug form, anthraquinone [116]. The CIA strategy effectively improves the solubility, bioavailability, and light-response efficiency of hydrophobic photo-sensitizing drugs, offering new ideas for light-controlled drug delivery systems.

**Figure 10:**
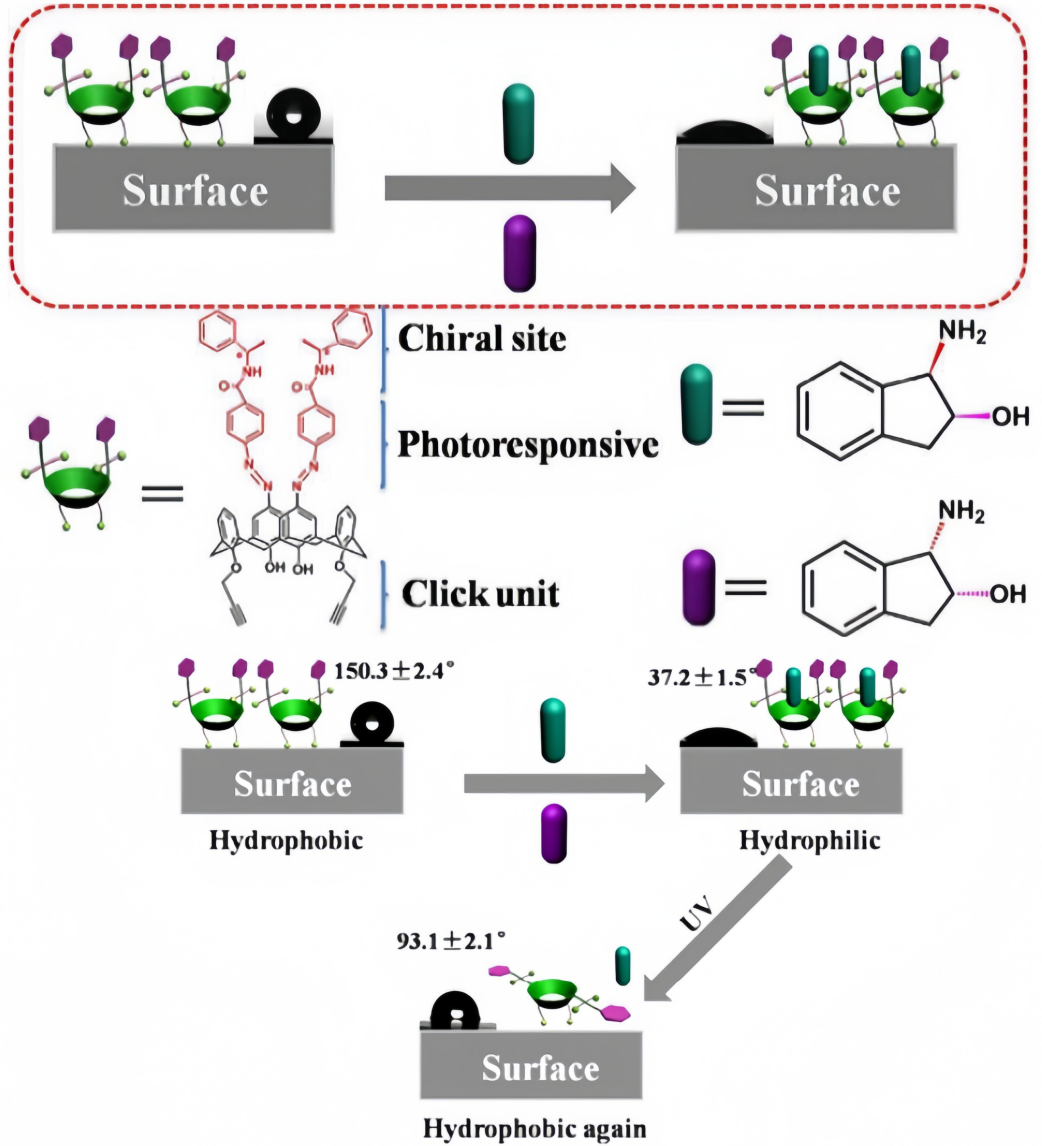
Schematic illustration of drug release controlled by the photo-responsive macroscopic switch based on FC4AD [117]. Figure 10 was used with permission of The Royal Society of Chemistry, from [117] (“A photo-responsive macroscopic switch constructed using a chiral azo-calix[4]arene functionalized silicon surface” by H. Pang et al., *Chem. Commun.*, vol. 54, issue 24, © 2018); permission conveyed through Copyright Clearance Center, Inc. This content is not subject to CC BY 4.0.

In 2018, Sun and colleagues designed and synthesized a chiral CA named chiral azo-calix[4]arene derivative (FC4AD) (Figure 10) [117]. Its interaction with enantiomers of aminoindanol in solution was investigated. It was found that FC4AD has high selective binding and release for (1*R*,2*S*)-1-amino-2-indanol, forming a 1:1 complex. *S*-phenethylamine was used as a chiral ligand, which was modified on the upper rim of calixarene through hydrogen bonding. An azobenzene group was introduced as the photo-regulating part at the upper edge of calixarene. The alkyne at the lower edge of FC4AD was used to form self-assembled monolayers (SAMs) on a silicon surface through Cu-catalyzed click chemistry to construct a photo-responsive macroscopic switch. This switch can achieve photo-controlled chiral reversible recognition of (1*R*,2*S*)-1-amino-2-indanol through changes in contact angle, and it holds promise for applications in chiral drug controlled release and other biotechnological fields. Further research can be conducted on the stability and performance of this switch under different environmental conditions. Its practical application in chiral drug controlled release can be expanded. Exploration of its interactions with other biomolecules can also be carried out to develop more applications in biosensing and biotechnology.

**Figure 11:**
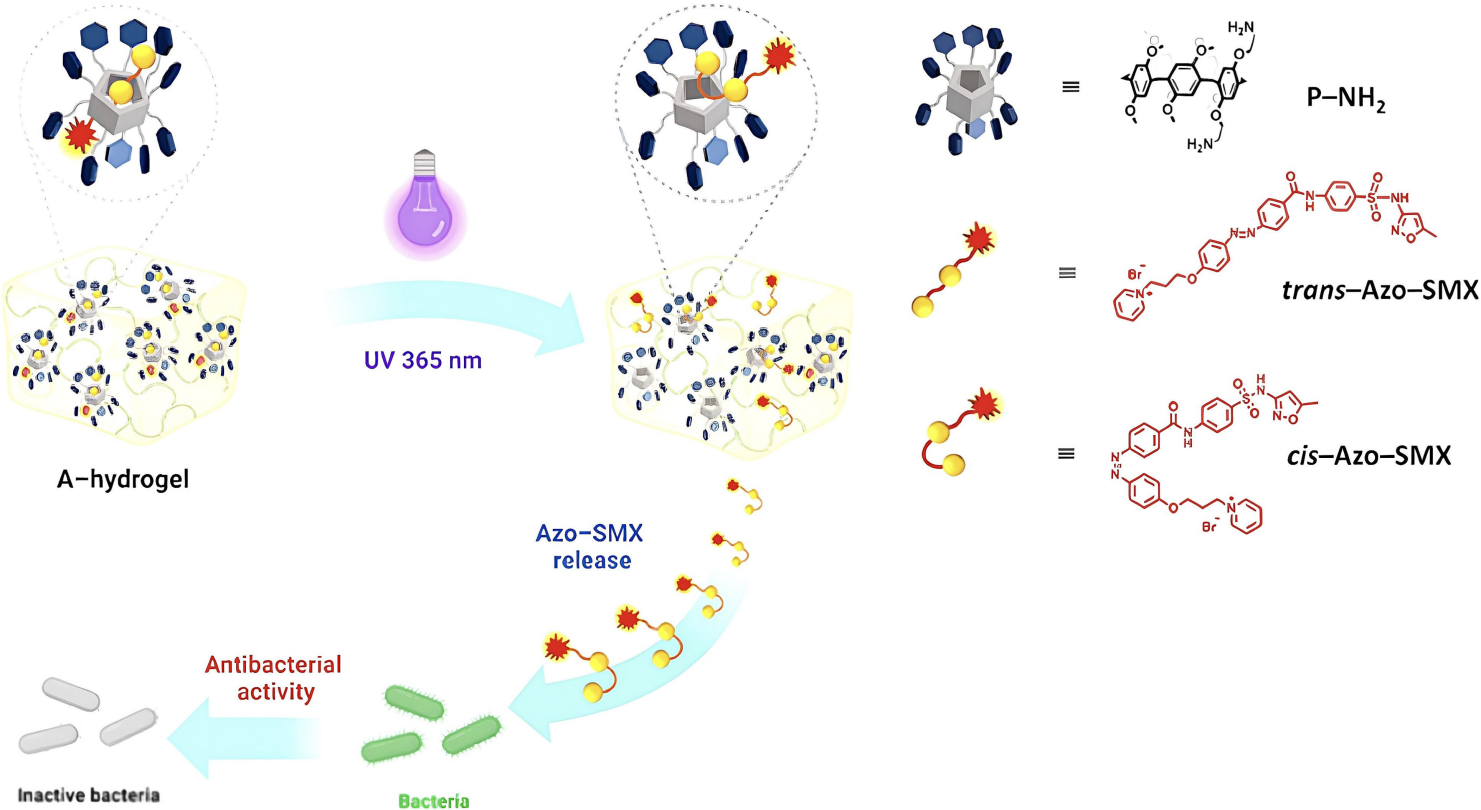
Schematic illustration of the formation process of Azo-SMX and its photoisomerization reaction under 365 nm UV light, transitioning from the trans to the cis form, which triggers its release from the inclusion complex [118]. Figure 11 was adapted with permission from [118], Copyright 2024 American Chemical Society. This content is not subject to CC BY 4.0.

In 2024, Thongnuek and coworkers crafted a PA5-linked gelatin hydrogel incorporating the Azo-SMX drug (referred to as A-hydrogel), aiming to achieve light-regulated uptake and delivery of antibiotics (Figure 11) [118]. Azobenzene, when subjected to particular light wavelengths, displays reversible switching between its *trans* and *cis* forms, a molecular behavior well-documented in light-responsive systems. Initially, Azo-SMX, bearing an azobenzene unit, exists in a trans form and creates a stable complex with the PA5 component within the hydrogel. Upon exposure to 365 nm UV light, Azo-SMX transitions from the *trans* to the *cis* state, prompting its release from the complex. Research on antibacterial efficacy has confirmed that the modified Azo-SMX antibiotic is effective against a range of microorganisms, including both Gram-positive and Gram-negative bacteria.

#### 2.3 Enzyme-responsive controlled release

Elevated enzyme levels often mark the microenvironment of many pathological tissues, as well as intracellular and cellular compartments. These enzymes can serve as effective triggers for drug release in stimulus-responsive systems. Owing to the specificity of enzymatic reactions, enzyme-responsive systems can act as targeted drug-delivery vehicles. Proteases such as esterases and ureases are particularly overexpressed in cancerous tissues. Thus, incorporating specific substrate groups or sequences can enhance drug release. Among the various stimuli used to control responsive assemblies, enzymatic methods offer several benefits, including biocompatibility, high efficiency, specificity, and mild reaction conditions.

**Figure 12:**
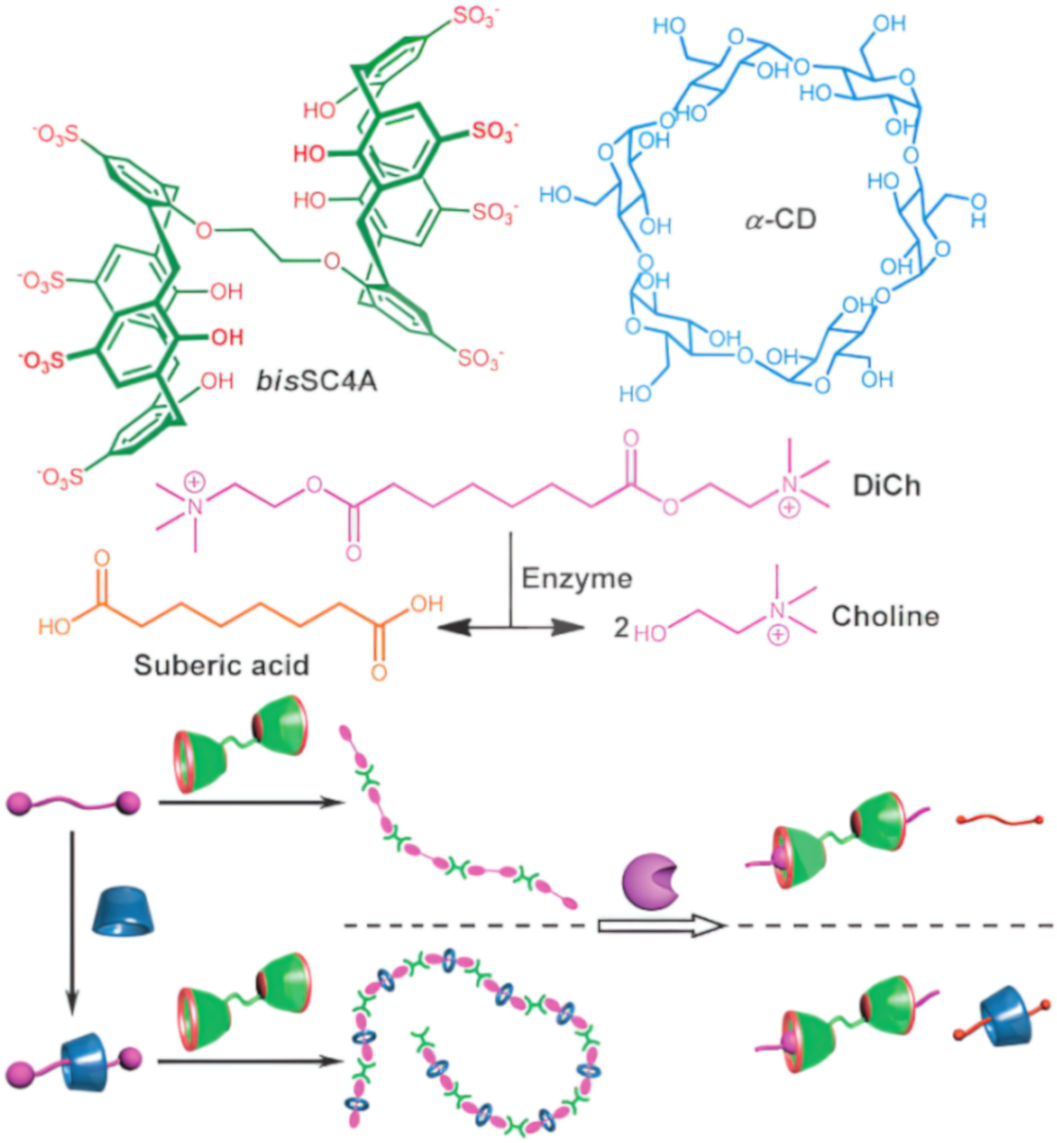
Schematic illustration of the enzyme-responsive behavior of supramolecular polymers [95]. Figure 12 was used with permission of The Royal Society of Chemistry, from [95] (“Enzyme-responsive supramolecular polymers by complexation of bis (*p*-sulfonatocalixarenes) with suberyl dicholine-based pseudorotaxane” by Guo, D.-S. et al., *Chem. Commun.*, vol. 49, © 2013); permission conveyed through Copyright Clearance Center, Inc. This content is not subject to CC BY 4.0.

**Figure 13:**
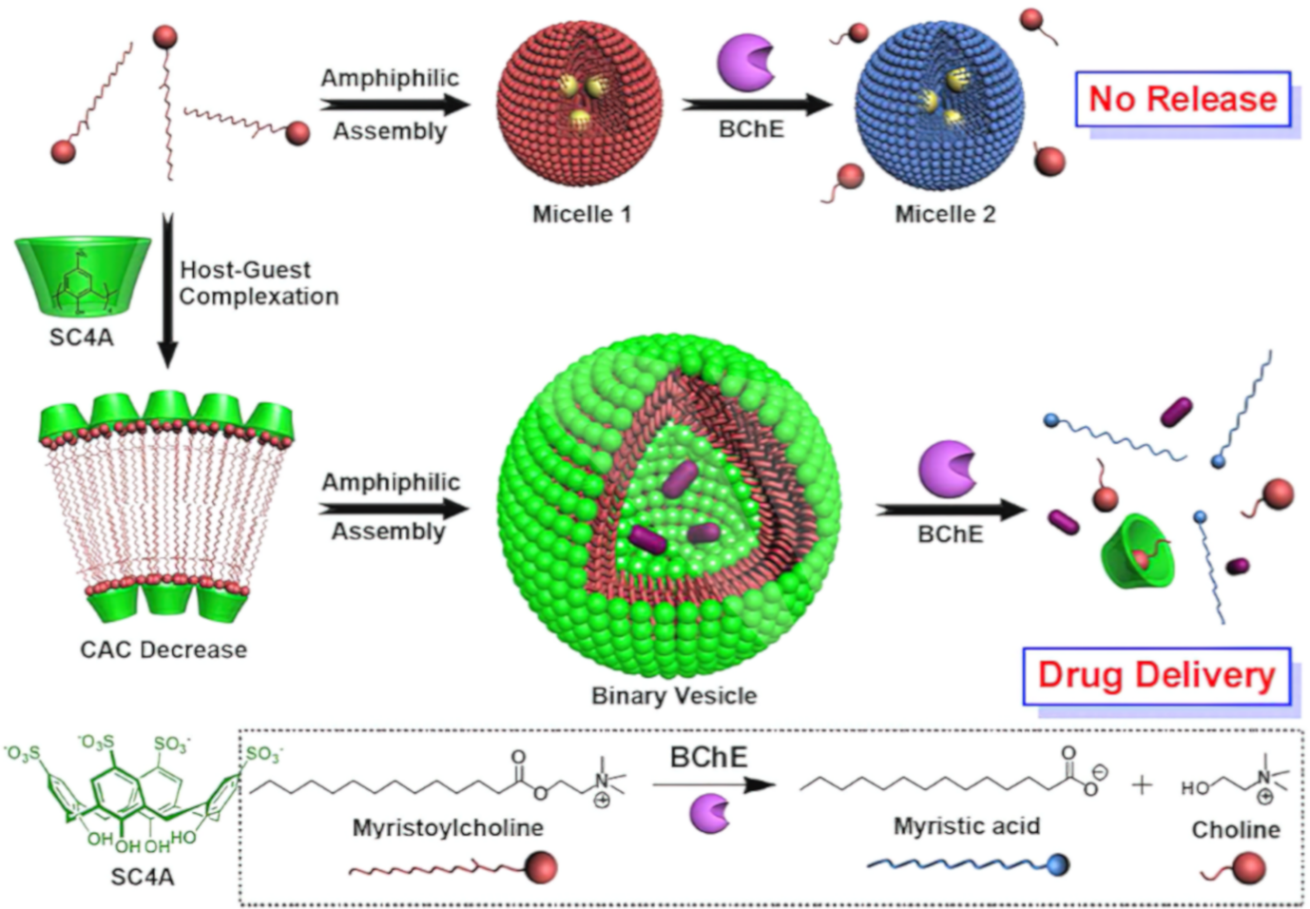
Schematic illustration of the amphiphilic assembly of SC4A and its enzyme-responsive applications [119]. Figure 13 was reprinted with permission from [119], Copyright 2012 American Chemical Society. This content is not subject to CC BY 4.0.

Liu and colleagues reported an enzyme-responsive supramolecular ternary polymer (DiCh@a-CD-bisSC4A) (Figure 12) [95]. The supramolecular polymer with suberyl dicholine (DiCh) as the axle, with α-CD as the thread wheel and bisSC4A macrocycles as the iterative end cap units. Due to the hydrolysis of DiCh by cholinesterase, the formed supramolecular polymer assembly can be dispersed through enzymatic reactions. They successfully prepared a novel supramolecular ternary polymer DiCh@a-CD-bisSC4A and binary polymers DiCh@bisSC4A Compared to binary DiCh@bisSC4A, ternary DiCh@a-CD-bisSC4A has not only a larger polymer size but also a better size distribution and topological structure due to the integration of quasi rotaxanes with supramolecular polymers. Introducing α-CD onto DiCh effectively adjusts the flexibility of the spacer group, which in turn enhances supramolecular polymerization. Despite DiCh being captured by both α-CD and bisSC4A, the resultant assembly retains enzyme responsiveness, attributed to the dynamic equilibrium inherent in non-covalent interactions. Building on biocompatibility studies, they also developed an enzyme-responsive supramolecular vesicle for treating Alzheimer's disease. This vesicle is assembled via the host–guest interaction between SC4A and myristoyl choline (Figure 13) [119]. The host–guest complexation between SC4A and myristoylcholine is stabilized by the electrostatic interaction between the negatively charged sulfonate groups and the positively charged quaternary ammonium groups. The interaction between the host and guest reduces the critical aggregation concentration (CAC) to form binary vesicles, with the hydrophobic alkyl chains of myristoylcholine packing together and the inner and outer surfaces composed of the hydrophilic phenolic OH groups of SC4A exposed to the aqueous solution. Subsequently, cholinesterase (acetylcholinesterase (AChE) and butyrylcholinesterase (BChE)) specifically converts myristoylcholine into myristic acid and choline [120]. SC4A binds to the non-amphiphilic choline but not to the amphiphilic myristic acid, leading to the disassembly of the composite vesicles. Currently, the clinical treatment of Alzheimer's disease is mainly based on cholinesterase inhibitors, such as tacrine. Therefore, this vesicle system may be suitable for the controlled release of Alzheimer's disease drugs.

**Figure 14:**
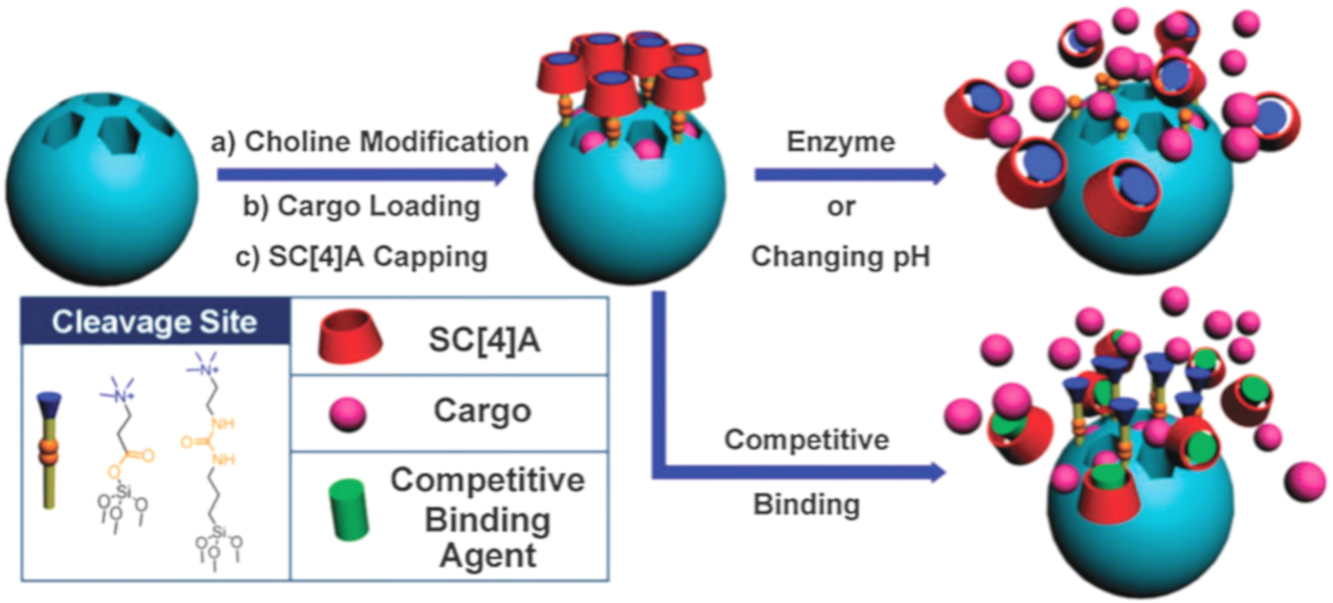
Stimuli-responsive nanovalves based on MSNs and choline-SC4A[2]pseudorotaxanes, MSN-C1 with ester-linked stalks and MSN-C2 with urea-linked stalks [121]. Figure 14 was used with permission of The Royal Society of Chemistry, from [121] (“Enzyme-responsive supramolecular nanovalves crafted by mesoporous silica nanoparticles and choline-sulfonatocalix [4] arene [2] pseudorotaxanes for controlled cargo release” by Sun, Y.-L. et al., *Chem. Commun.*, vol. 49, issue 79, © 2013); permission conveyed through Copyright Clearance Center, Inc. This content is not subject to CC BY 4.0.

Extensive research has been conducted on MSN carriers fitted with supramolecular nano valves. Yang and co-workers [121] designed an enzyme-responsive supramolecular nanovalve composed of MSNs and choline sulfonatocalixarene[2]pseudorotaxane. Two different choline derivatives with distinct structures and lengths (Figure 14) were grafted onto the pores of MSNs via ester or urea linkages, serving as enzyme-cleavable sites. Negatively charged SC4A macrocycles were introduced to envelop the choline stems on the surface of MSN-NPs through host–guest interactions, forming pseudorotaxanes as the mobile/cleavable components of the nanovalves. It was experimentally demonstrated that esterase could selectively activate the ester-linked nanovalve (MSN-C1), while urease could selectively activate the urea-linked nanovalve (MSN-C2). This innovative enzyme-activated method provides inherent biocompatibility, mild reaction conditions, high efficiency, and specificity for the degradation of supramolecular polymers.

#### 2.4 Hypoxia-responsive controlled release

In the last few years, notable strides have been achieved in boosting the targeting accuracy and clinical utility of drug release mechanisms. Tumor cells are characterized by unique attributes, such as acidity, hypoxia, and altered metabolism, which set them apart from healthy cells. These distinct features underscore the importance of focusing on the tumor microenvironment to develop supramolecular systems that are highly specific and capable of responding to multiple stimuli. These advanced systems aim to target and penetrate tumor cells effectively, mitigate drug resistance caused by tumor cell uptake, and ultimately enhance clinical efficacy.

Guo and associates developed hypoxia-responsive molecular carriers, namely carboxylated or sulfonated azo-calix[4/5]arenes [122–123]. These CAs, utilizing their azo groups that are easily reduced in low-oxygen settings, provide the potential for tumor-targeted drug release while reducing side effects. The azo-calix[4/5]arenes have demonstrated strong binding capabilities with a range of chemotherapeutic drugs, which underscores their potential as supramolecular drug carriers. They have verified the efficacy of this hypoxia-targeted therapy through both in vitro and in vivo experiments, and the carrier has made significant progress in the field of hypoxia-targeted drug delivery. This section highlights several recent studies on stimulus-responsive drug release within hypoxic tumor environments.

**Figure 15:**
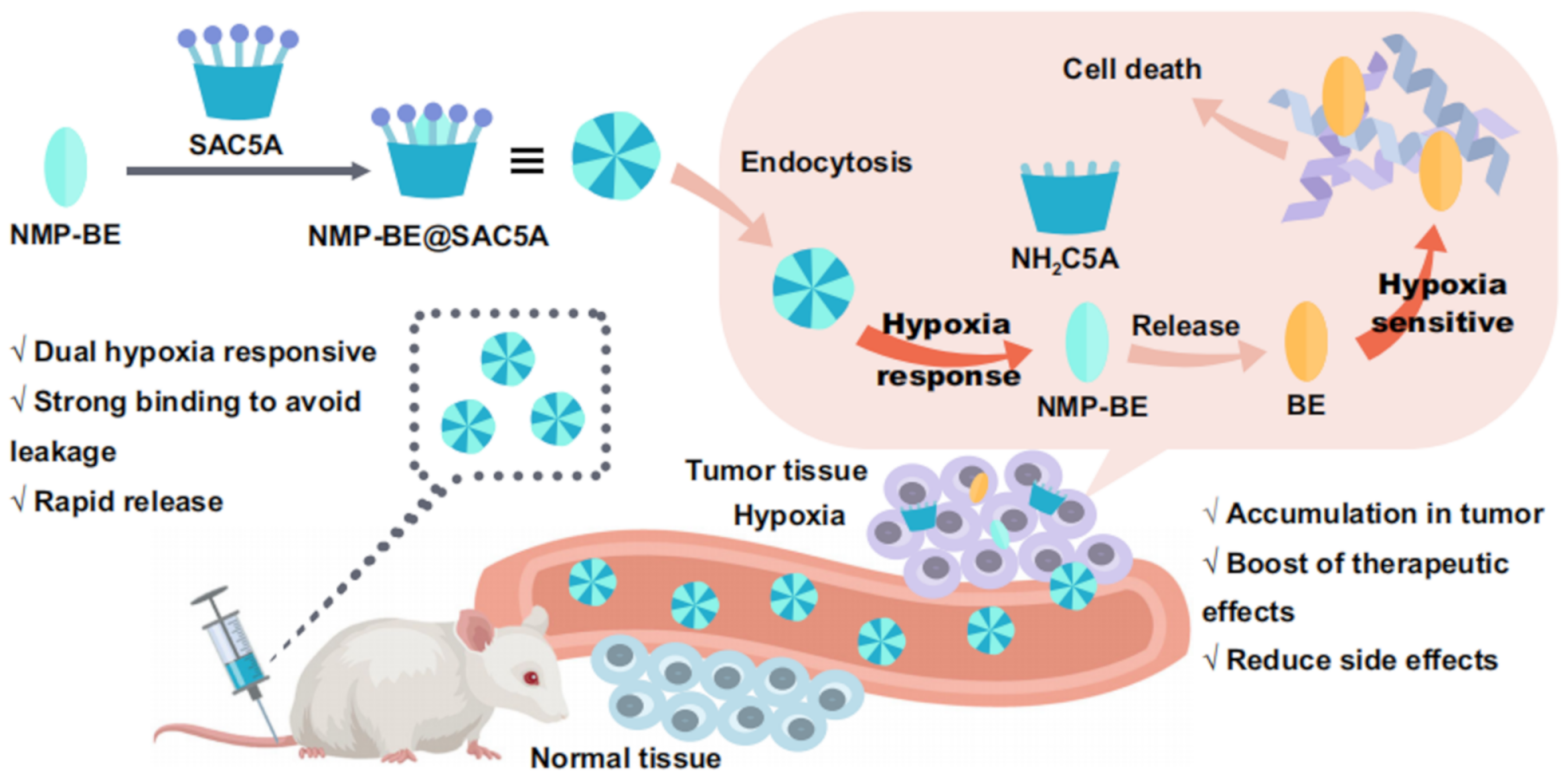
A schematic diagram showing the construction of a supramolecular system by host–guest interaction between SAC5A and the NMP-BE prodrug under hypoxic conditions [123]. Figure 15 was reproduced from [123] (© 2023 J.-S. Guo et al., published by Springer Nature, distributed under the terms of the Creative Commons Attribution 4.0 International License, https://creativecommons.org/licenses/by/4.0).

Among anti-cancer drugs, BE-43547A2 (BE) exhibits high cytotoxicity against hypoxic pancreatic cancer cells, which is in line with the current hypoxic tumor environment, making it a promising candidate for hypoxia-targeted therapies. The Guo group modified calixarene to obtain sulfonated azo-calix[5]arene (SAC5A), and then combined the BE prodrug (NMP-BE) with the SAC5A to create a supramolecular complex, NMP-BE@SAC5A, that is responsive to dual hypoxia signals (Figure 15) [123]. The azo group in SAC5A endows it with hypoxia responsiveness, allowing it to enhance the accumulation of NMP-BE in tumors under hypoxic conditions in cancer cells. Upon intracellular release in cancer cells, NMP-BE releases the hypoxia-sensitive toxin BE, achieving the dual hypoxia-responsive therapeutic goal through host–guest interactions. Due to the specific cellular uptake and discharge process, NMP-BE@SAC5A effectively suppresses the proliferation of pancreatic cancer in a mouse model with human tumor cells at minimal concentrations, while avoiding adverse effects throughout the body. In the future, it could be attempted to combine it with more anti-tumor drugs and apply it in clinical settings.

**Figure 16:**
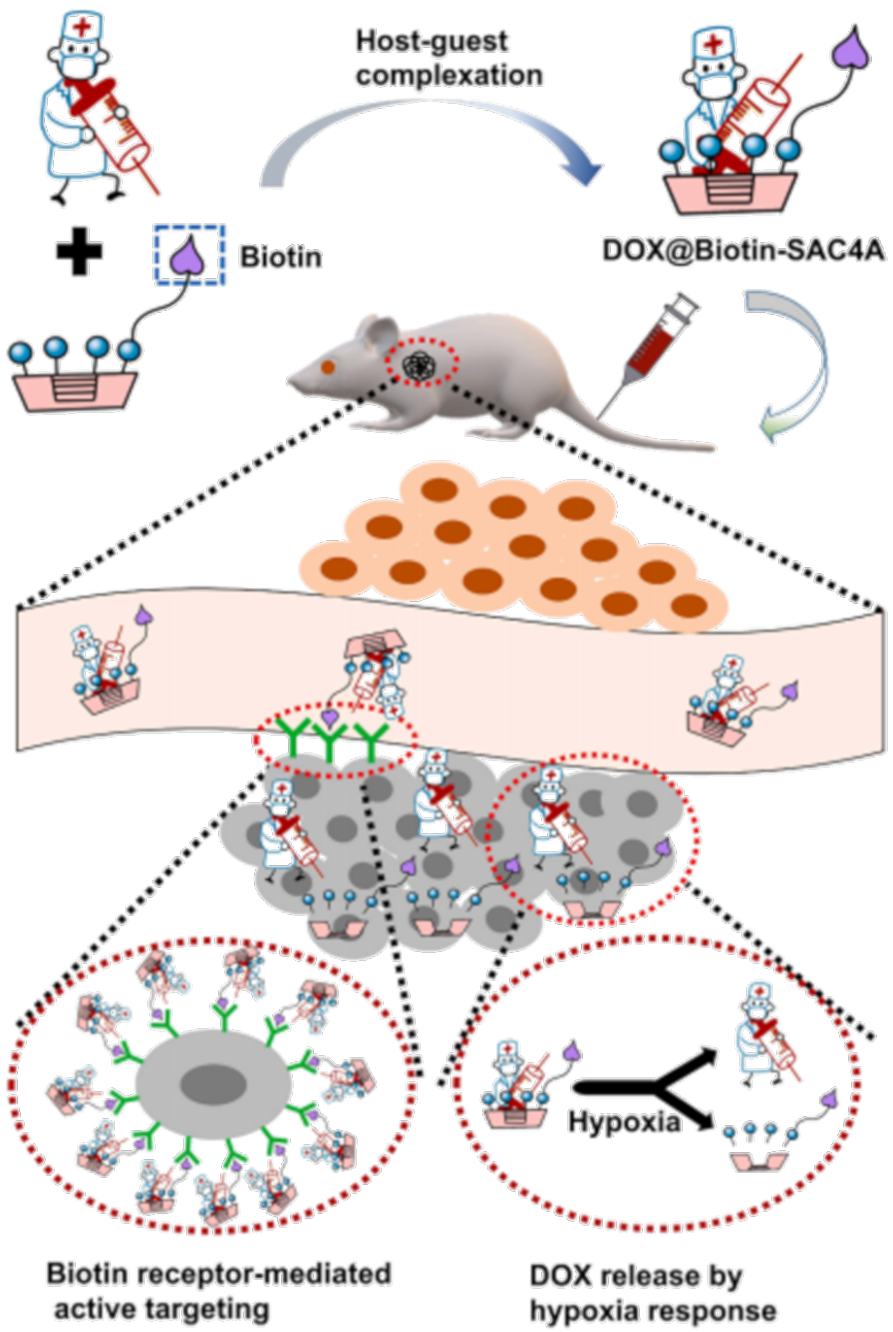
A schematic diagram showing the formation of the host–guest complex DOX@Biotin-SAC4A by biotin modification of SAC4 and the release of DOX under hypoxic conditions in mice [124]. Figure 16 was reprinted from [124], *Journal of Controlled Release*, vol. 368, by Chen, M.-M.; Tang, X.; Li, J.-J.; Chen, F.-Y.; Jiang, Z.-T.; Fu, R.; Li, H.-B.; Hu, X.-Y.; Geng, W.-C.; Guo, D.-S., “Active targeting tumor therapy using host–guest drug delivery system based on biotin functionalized azocalix [4] arene”, pages 691–702, Copyright (2024), with permission from Elsevier. This content is not subject to CC BY 4.0.

In addition to the aforementioned SAC5A that can respond to stimuli under hypoxic conditions, they also synthesized Biotin-SAC4A by modifying SAC4A with biotin. (Figure 16) [124]. Monocarboxylated azocalixarene and aminated biotin were synthesized. Subsequently, using a coupling agent, the carboxyl group of the azocalixarene was modified through amidation to obtain biotin-modified SAC4A. The formation of amide bonds and azo linkages deepened the cavity and enhanced the interaction strength with drugs. The biotin on the upper rim endows biotin-SAC4A with the ability to actively target tumor cells based on the interaction between biotin and biotin receptors, thereby improving antitumor efficacy. DOX@biotin-SAC4A exhibited high cytotoxicity against cancer cells but low toxicity to normal cells. They further combined Biotin-SAC4A with DOX and injected it into mice. The experimental results showed that Biotin-SAC4A could firmly bind DOX and promote its release under hypoxic conditions.

**Figure 17:**
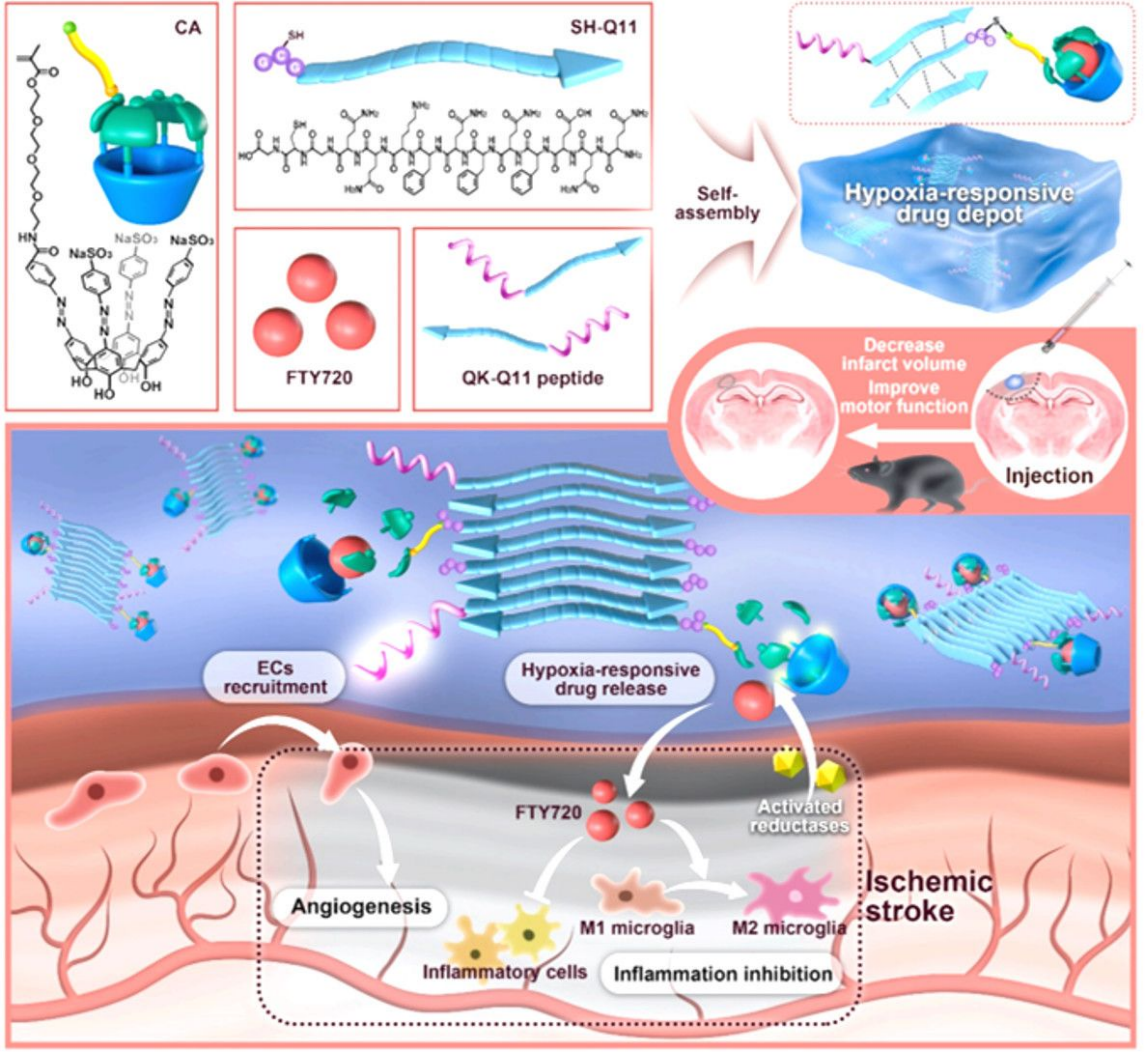
A schematic diagram showing the self-assembly of CA4 into a hypoxia-responsive peptide hydrogel, which achieves targeted release of CY5-DM or FTY720 in vitro [125]. Figure 17 was reprinted from [125], *Nano Today*, vol. 54, by Zheng, W.; Yao, S.-Y.; Hu, H.; Chen, X.; Qian, Z.; Liu, W.; Zhu, Y.; Mao, Z.; Guo, D.-S.; Gao, C., “Hypoxia-responsive calixarene-grafted self-assembled peptide hydrogel for inflammation suppression in ischemic stroke”, article no. 102064, Copyright (2023), with permission from Elsevier. This content is not subject to CC BY 4.0.

In 2024, Guo and colleagues developed a SAC4A grafted self-assembled peptide hydrogel for hypoxia-responsive controlled drug release in anti-inflammatory treatment of ischemic stroke (Figure 17) [125]. Following PBS induction, the peptide hydrogel was tailored to exhibit a rheological modulus and shear-thinning behavior similar to that of brain tissue. The hydrogel’s hypoxia-responsive properties were validated through three different systems: sodium dithionite (SDT), rat liver microsomes, and the oxygen-glucose deprivation (OGD) model. Under hypoxic conditions, the hydrogel achieved targeted release of cyanine 5-dimethyl (CY5-DM) or fingolimod (FTY720) in vitro. Upon local administration, the hypoxia-responsive self-assembling peptide hydrogel resulted in improved motor function and reduced inflammation in vivo.

**Figure 18:**
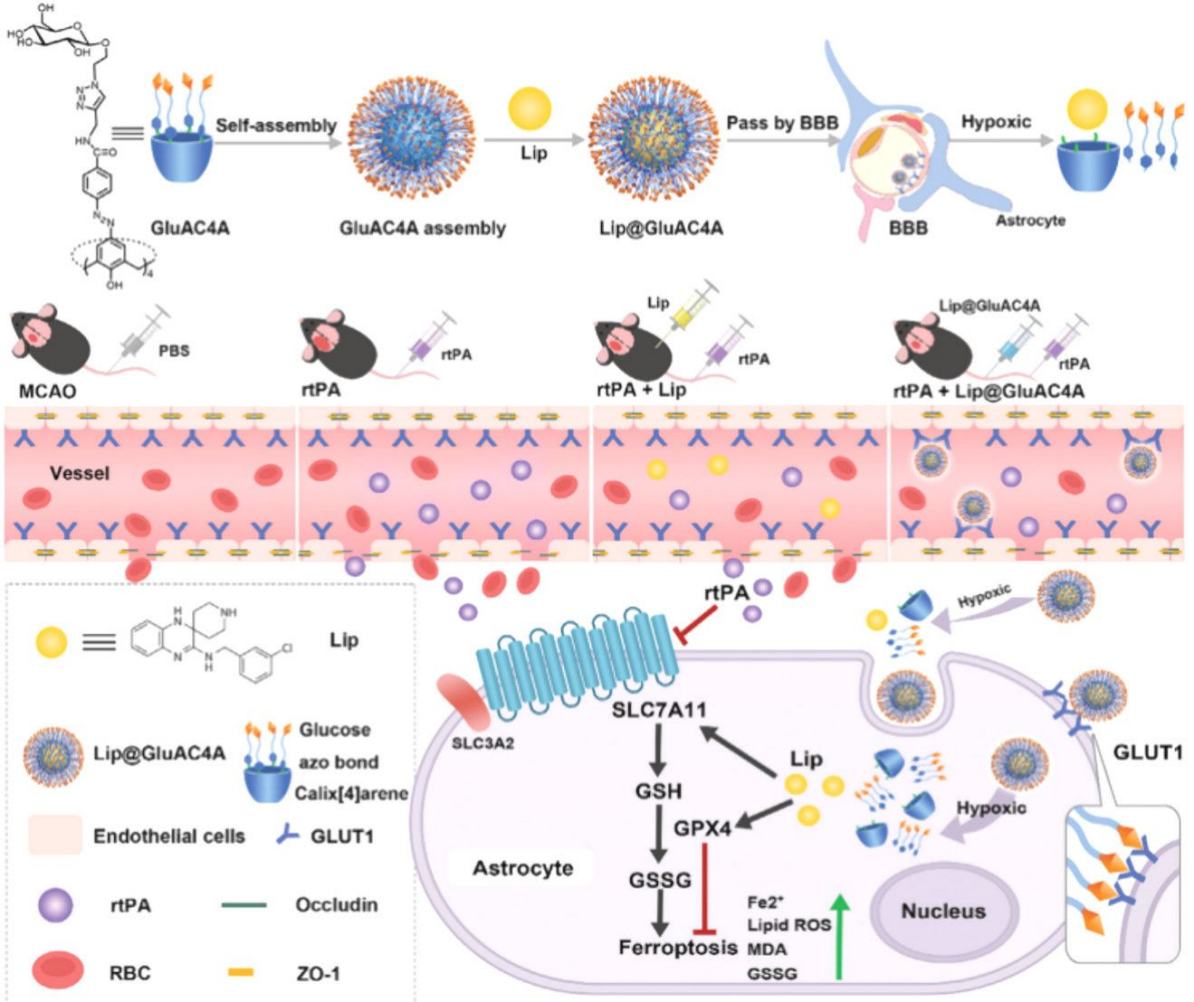
Schematic illustration of the formation process of Lip@GluAC4A and the release of Lip under hypoxic conditions in mice [126]. Figure 18 was reproduced from [126] (© 2024 Y.-Q. Geng et al., *Advanced Science*, published by Wiley-VCH GmbH, distributed under the terms of the Creative Commons Attribution 4.0 International License, https://creativecommons.org/licenses/by/4.0).

Recently, Geng and colleagues created additional binding sites by modifying the upper rim of CA4 with glucose and enhanced the overall solubility and biocompatibility of the azo-calixarene structure, designing it as a drug carrier (Figure 18) [126]. This carrier binds to the ferroptosis inhibitor liproxstatin-1 (Lip) and releases it selectively in hypoxic environments. This mechanism aims to counteract the side effects associated with intravenous thrombolysis using recombinant tissue-type plasminogen activator (rtPA) for treating ischemic stroke. The interaction between GluAC4A and Lip results in a significant improvement in Lip’s solubility. The glucose modification on the upper rim of GluAC4A serves multiple functions: it expands the calixarene cavity, creates additional binding sites, and enhances the overall solubility and biocompatibility of the azo-calixarene structure. The azo moiety in GluAC4A imparts hypoxia responsiveness, enabling the targeted release of Lip specifically at ischemic sites. GluAC4A effectively promotes drug accumulation in the brain. Moreover, the Lip@GluAC4A complex significantly reduces iron deposition, blood-brain barrier leakage, and neurological deficits that are commonly observed with rtPA treatment.

#### 2.5 Multi-responsive controlled release

The previous sections discussed the controlled drug release of single-stimulus-responsive systems, adopting a relatively simple perspective of a single stimulus-response. Multistimuli responsiveness also holds potential applications in terms of responsiveness and efficient drug release. One of the major challenges faced by researchers in this field has long been the difficulty of incorporating two or more functional groups simultaneously when constructing multi-stimuli-responsive systems. Supramolecular materials can also be designed to be responsive to multiple stimuli. In addition to those mentioned above, multi-stimulus responses have a broad application prospect in controlling drug release. For example, supramolecular peptide assemblies that naturally respond to pH or ion strength can be designed to include light-responsive elements or feature-specific enzyme substrates, thus utilizing two separate stimulus-response pathways.

In 2011, Liu and colleagues [127] created a multi-stimulus-responsive supramolecular bis-vesicle through the complexation of water-soluble calixarene (C4AS) with 1-dodecyl-1'-methyl-4,4'-bipyridinium (MVC12). The term "bis-vesicle" refers to a structure with two fused or nested vesicular domains, typically featuring shared membrane interfaces or segregated internal spaces. They subsequently loaded the anticancer drug DOX into the vesicle. The formation mechanism benefits from the rigid conical structure of C4AS, which significantly reduces the critical aggregation concentration of MVC12 by 1000 times. This bis-vesicle structure possesses the following intelligent responsive characteristics: (1) temperature sensitivity originating from the enthalpy-driven nature of the host–guest interaction; (2) redox responsiveness due to the reversible electron transfer of MVC12; (3) competitive complexation making the system sensitive to cyclodextrins. Experiments have confirmed that this carrier can efficiently encapsulate DOX and trigger drug release through multiple stimuli, demonstrating good potential for anticancer therapy in vitro. This intelligent delivery system, integrating temperature, redox, and molecular recognition responses, provides important ideas for the development of new cancer treatment strategies.

**Figure 19:**
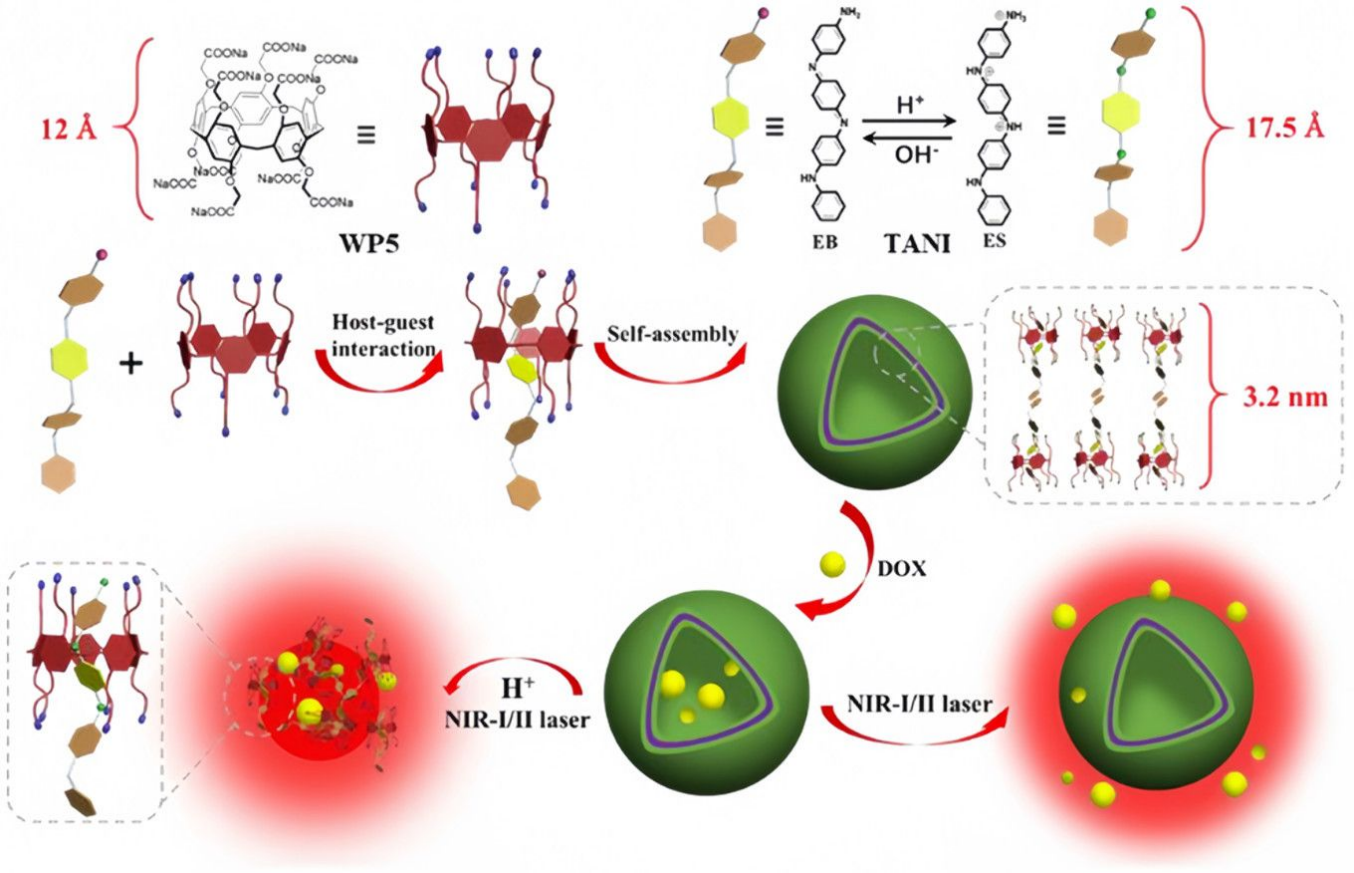
Schematic illustration of the construction of a supramolecular vesicle based on the host–guest complexation between WP5 and TANI, and the release of DOX under acidic conditions or NIR irradiation [128]. Figure 19 was reprinted from [128], *Chemical Engineering Journal*, vol. 403, by Chen, X.; Wang, Z.; Sun, X.; Han, Y.; Huang, Y.; Xi, J.; Bian, X.; Han, J.; Guo, R., “Photothermal supramolecular vesicles coassembled from pillar [5] arene and aniline tetramer for tumor eradication in NIR-I and NIR-II biowindows”, Copyright (2020), with permission from Elsevier. This content is not subject to CC BY 4.0.

In 2021, Han et al. explored the interactions WP5 and aniline tetramer (TANI) as host and guest molecules (Figure 19) [128]. The WP5⊃TANI complex is capable of self-assembling into supramolecular vesicles. WP5 has a large hydrophobic cavity that can accommodate TANI, and the interaction between WP5 and TANI leads to the formation of an amphiphilic complex. The amphiphilicity of the WP5-TANI complex, the π–π stacking interactions between the two components, and the high hydrophobicity of TANI promote the host–guest interactions between WP5 and TANI, leading to the further formation of supramolecular vesicles by the WP5⊃TANI complex. These vesicles are non-toxic to normal cells but exhibit toxicity towards cancer cells. The WP5⊃TANI supramolecular vesicles can effectively encapsulate the anticancer drug DOX. Both the acidic microenvironment at the tumor site and photothermal effects can induce the release of DOX. DOX delivered by these vesicles, in combination with NIR light irradiation, can significantly suppress tumor growth.

**Figure 20:**
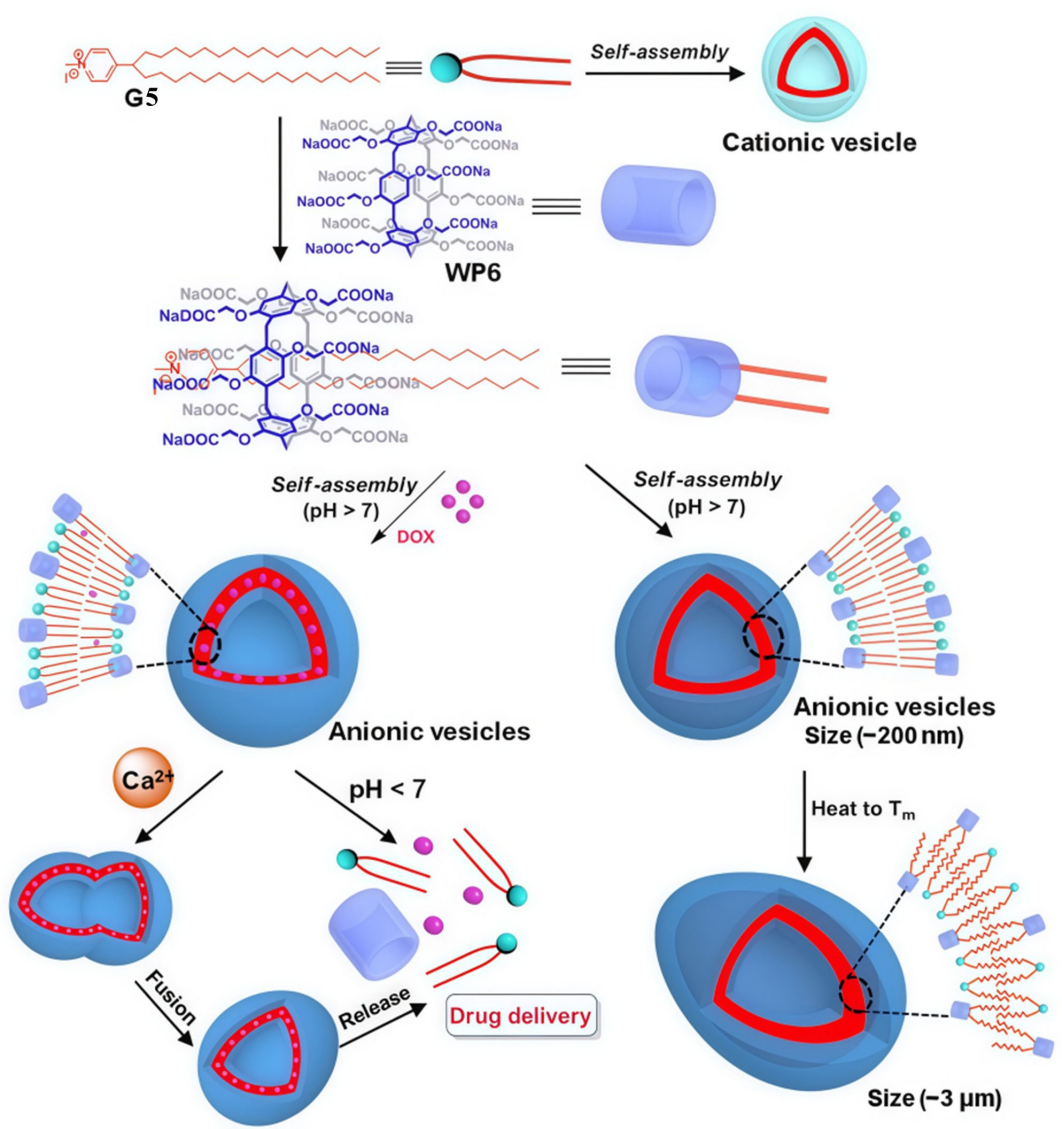
Schematic illustration of WP6 self-assembly at pH > 7, and the stimulus-responsive drug release behavior under varying conditions of Ca^2+^ concentration, pH, and temperature [107]. Figure 4 was adapted with permission from [107], Copyright 2014 American Chemical Society. This content is not subject to CC BY 4.0.

Wang and colleagues [107] designed a supramolecular binary vesicle system based on WP6 and SAINT molecules (G5), which exhibits multiple stimulus-responsive characteristics to pH, Ca^2+^ and temperature (Figure 20). WP6 and G5, as amphiphilic molecules containing pyridine groups and alkyl chains, form supramolecular amphiphiles through host−guest interactions, with the assembly process driven by both hydrophobic and electrostatic interactions. The vesicles can efficiently encapsulate drug molecules such as calcein and DOX, and trigger their release under acidic conditions or Ca^2+^ stimulation. The fusion process of WP6⊃G5 vesicles can be divided into three stages: First, Ca^2+^ binds to the carboxyl groups on the outer surface of the WP6⊃G5 vesicles. Second, the binding of Ca^2+^ to WP6 further induces vesicle aggregation. Third, vesicle fusion occurs. Moreover, temperature regulation can achieve reversible changes in vesicle size; heating forms stable vesicles with an increased internal cavity and a size of approximately 3 micrometers, providing a potential carrier for bioimaging applications. This intelligent vesicle system, which combines drug-controlled release and tunable structural properties, holds significant value in the fields of targeted drug delivery and biomedical applications.

**Figure 21:**
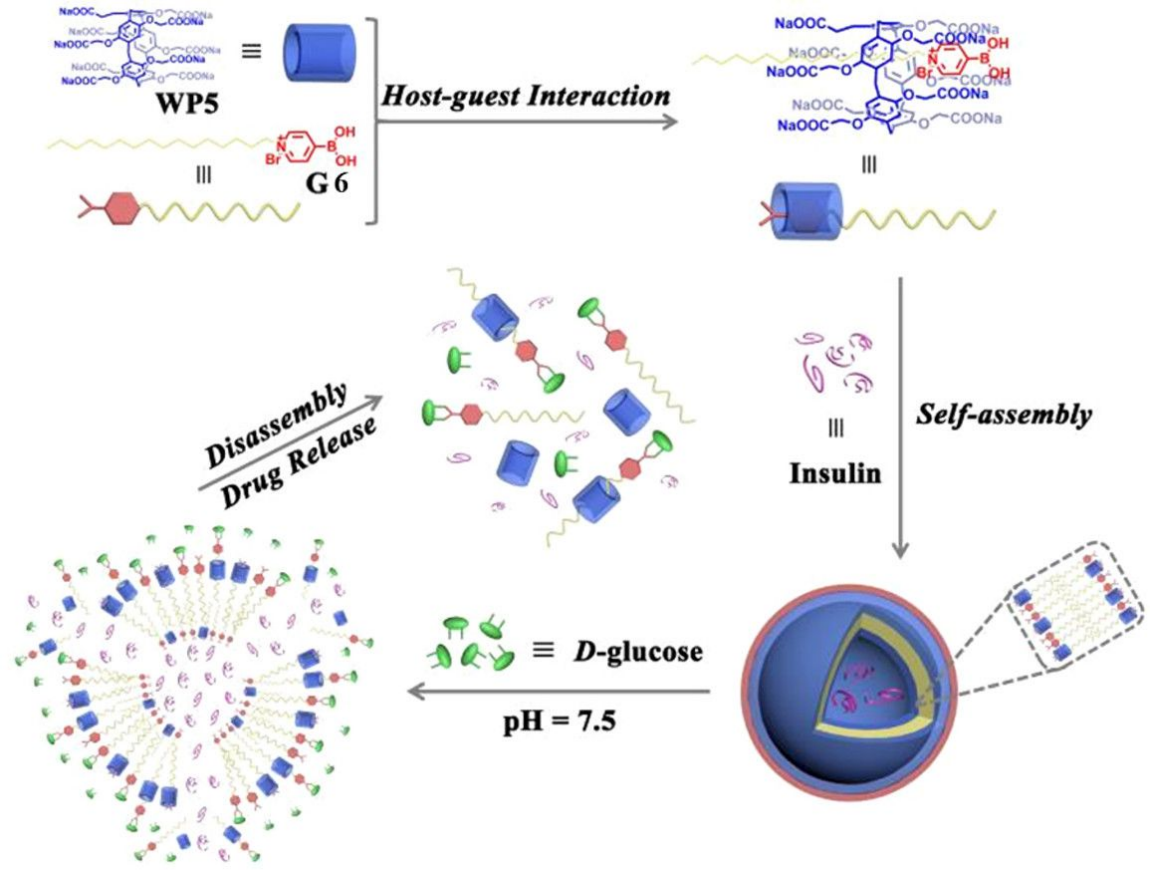
Schematic illustration of the formation of supramolecular vesicles based on the WP5⊃G super-amphiphile and their stimulus-responsive release [129]. Figure 21 was reproduced from [129], L. Gao et al., “Glucose-Responsive Supramolecular Vesicles Based on Water-Soluble Pillar[5]arene and Pyridylboronic Acid Derivatives for Controlled Insulin Delivery”, *Chem. Eur. J.*, with permission from John Wiley and Sons. Copyright © 2017 Wiley-VCH Verlag GmbH & Co. KGaA, Weinheim. This content is not subject to CC BY 4.0.

Subsequently, Wang and colleagues [129] in 2017 employed WP5 and pyridinium boronic acid derivatives (G6) to encapsulate insulin and achieve controlled release under physiological conditions (Figure 21). The host–guest binding affinity, which occurs in a 1:1 ratio, is primarily driven by synergistic electrostatic and hydrophobic interactions. The WP5⊃G6 supramolecular vesicles exhibit unique dual responsiveness to glucose and pH, originating from the specific binding of the pyridine-boronic acid motif to the cis-diol of ᴅ-glucose and the pH-sensitive solubility changes. Under physiological pH conditions (7.4), the vesicle structure rapidly dissociates upon the addition of ᴅ-glucose or when the pH is lowered to 6.0, as evidenced by the disappearance of the Tyndall effect and the absence of vesicular structures in TEM images. This response mechanism is attributed to: 1) competitive binding of glucose leading to the dissociation of the host–guest complex; and 2) protonation of the WP5 carboxyl groups under acidic conditions, forming insoluble precipitates. The system can efficiently encapsulate insulin and precisely release it in response to high glucose levels or acidic environments demonstrating excellent potential for diabetes therapy. This intelligent delivery system achieves on-demand drug release and provides new insights for the development of glucose-responsive insulin formulations.

**Figure 22:**
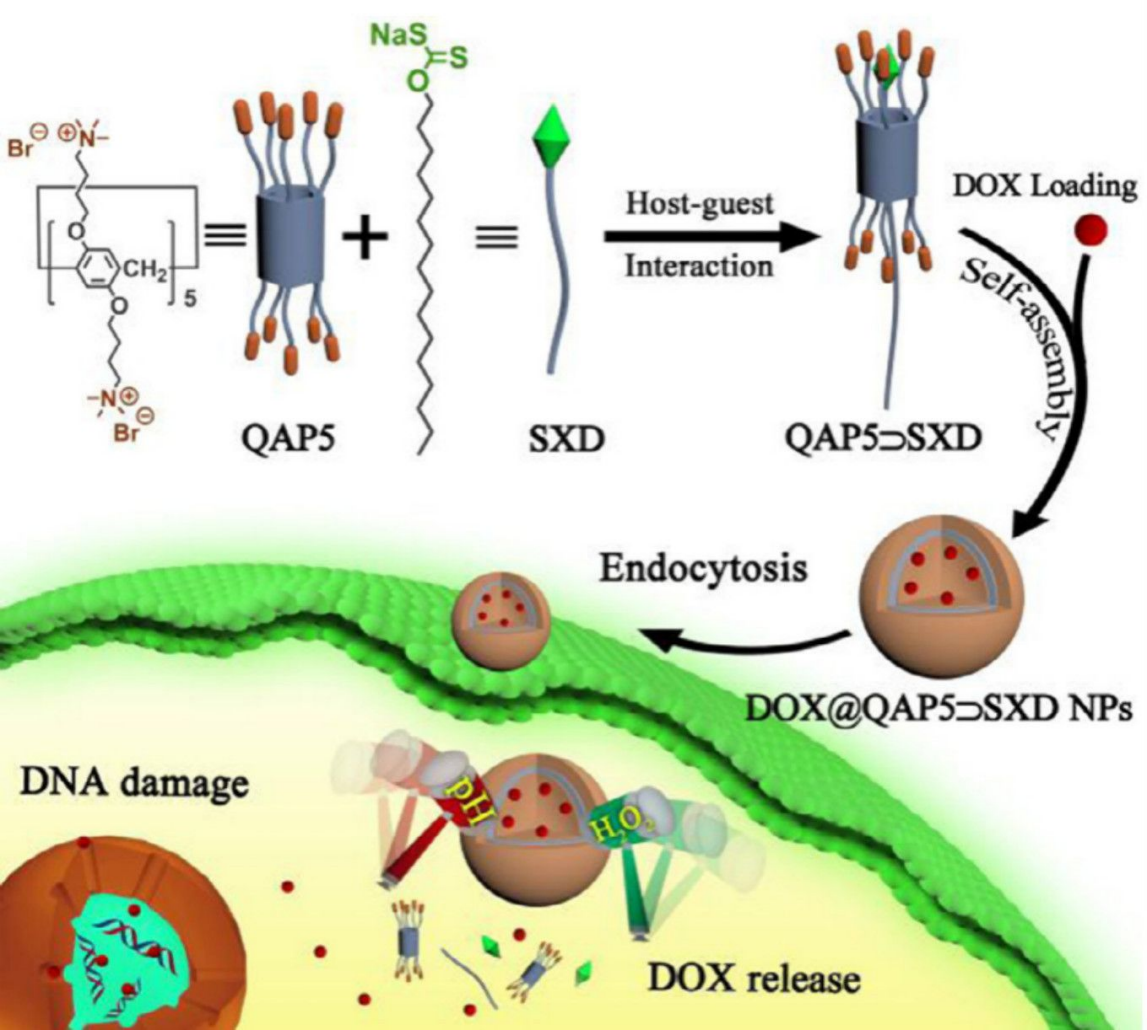
Schematic illustrations of the host–guest recognition of QAP5⊃SXD, the formation of the nanoparticles DOX@QAP5⊃SXD, and the stimulus-responsive release of DOX under pH and H₂O₂ conditions [130]. Figure 22 was reprinted from [130], *Chinese Chemical Letters*, vol. 33, by Shen, Z.; Ma, N.; Wang, F.; Ren, J.; Hou, C.; Chao, S.; Pei, Y.; Pei, Z., “pH-and H_2_O_2_-sensitive drug delivery system based on sodium xanthate: dual-responsive supramolecular vesicles from one functional group”, pages 4563-4566, Copyright (2022), with permission from Elsevier on behalf of Chinese Chemical Society and Institute of Materia Medica, Chinese Academy of Medical Sciences. This content is not subject to CC BY 4.0.

In 2022, Pei and collaborators unveiled a dual-stimuli-responsive supramolecular drug delivery system that relies on host–guest interactions, with sodium xanthate derivatives (SXD) serving as guest molecules and quaternary ammonium pillar[5]arene (QAP5) acting as host molecules (Figure 22) [130]. The hydrophobic SXD and the hydrophilic QAP5 interact through host–guest interactions driven by electrostatic forces to form the QAP5–SXD complex. The amphiphilic nature of QAP5–SXD enables its self-assembly into stable QAP5–SXD nanoparticles in aqueous solution, which are identified as vesicles by TEM. In this study, the xanthate group, which responds to both pH and hydrogen peroxide, shows great potential for the development of dual-responsive drug delivery systems. Using DOX as a model anticancer drug, they synthesized DOX@QAP5⊃SXD nanoparticles. Under conditions of low pH and high hydrogen peroxide concentration, SXD can rapidly decompose, triggering the disassembly of DOX@QAP5⊃SXD nanoparticles and the release of the encapsulated drug DOX.

**Figure 23:**
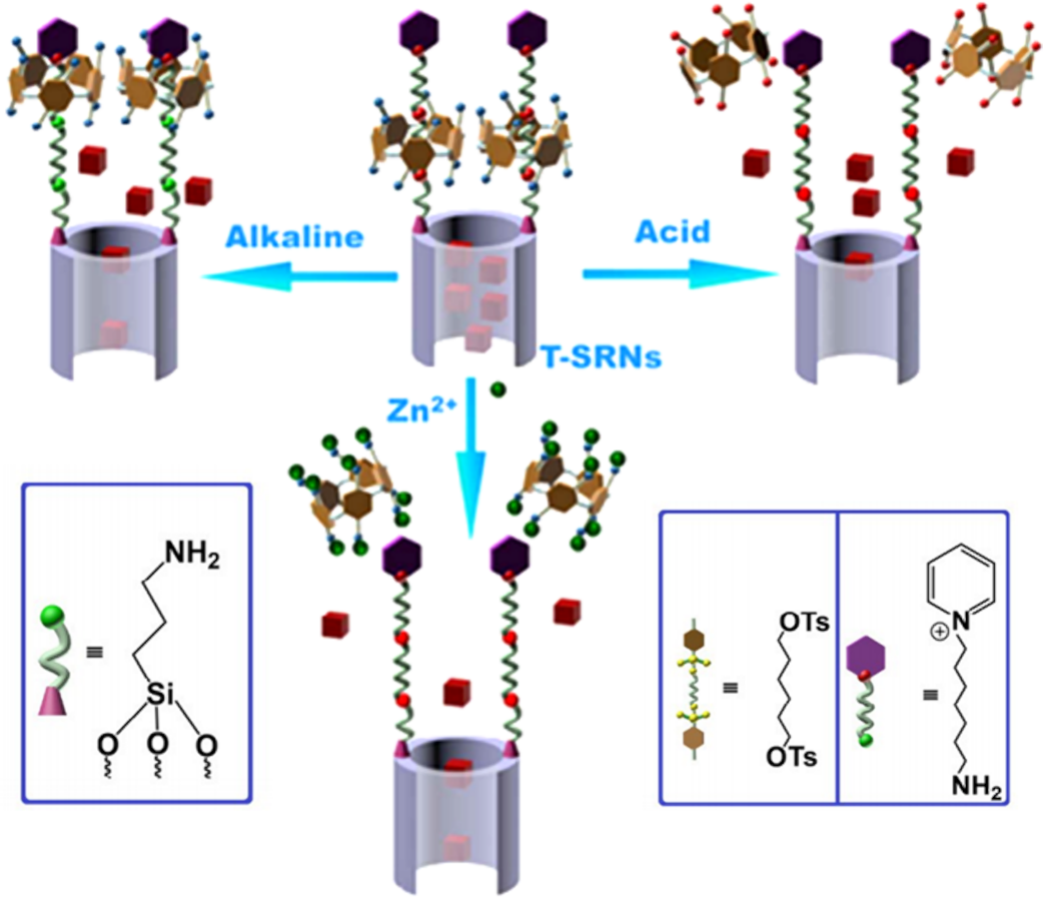
Schematic illustration of the activation of T-SRNs by acid, alkali, or Zn^2+^ stimuli to regulate the release of the model drug Rhodamine B (RhB) [132]. Figure 23 was used with permission of The Royal Society of Chemistry, from [132] (“Triple-stimuli-responsive nanocontainers assembled by water-soluble pillar [5] arene-based pseudorotaxanes for controlled release” by C. Ding et al., *J. Mater. Chem. B*, vol. 4, issue 16, © 2016); permission conveyed through Copyright Clearance Center, Inc. This content is not subject to CC BY 4.0.

Du and his team engineered MSN functionalized with dimethyl benzimidazolium (DMBI) and bipyridinium (BP) groups [131], as well as carboxylic acid-modified PA6 hydrocarbons (CPA6) to create multi-responsive controlled-release systems. CPA6 can rotate around DMBI or BP to form supramolecular nanovalves that encapsulate drugs within the MSN pores. The release of drugs is triggered by competitive binding at acidic pH or CPA6 de-threading, which then opens the nanovalves. In 2016, Fu and colleagues [132] designed and constructed an acid/base/Zn^2+^ stimulus-responsive controlled release system on the outer surface of a WP5-based bistable[2]pseudorotaxane (Figure 23). WP5 bonds with 1,6-hexane diamine (HDA) under neutral conditions, acting as a plug to block the retained cargo, and the two dissociate in acidic, basic, or Zn^2+^ environments, leading to controlled drug release.

**Figure 24:**
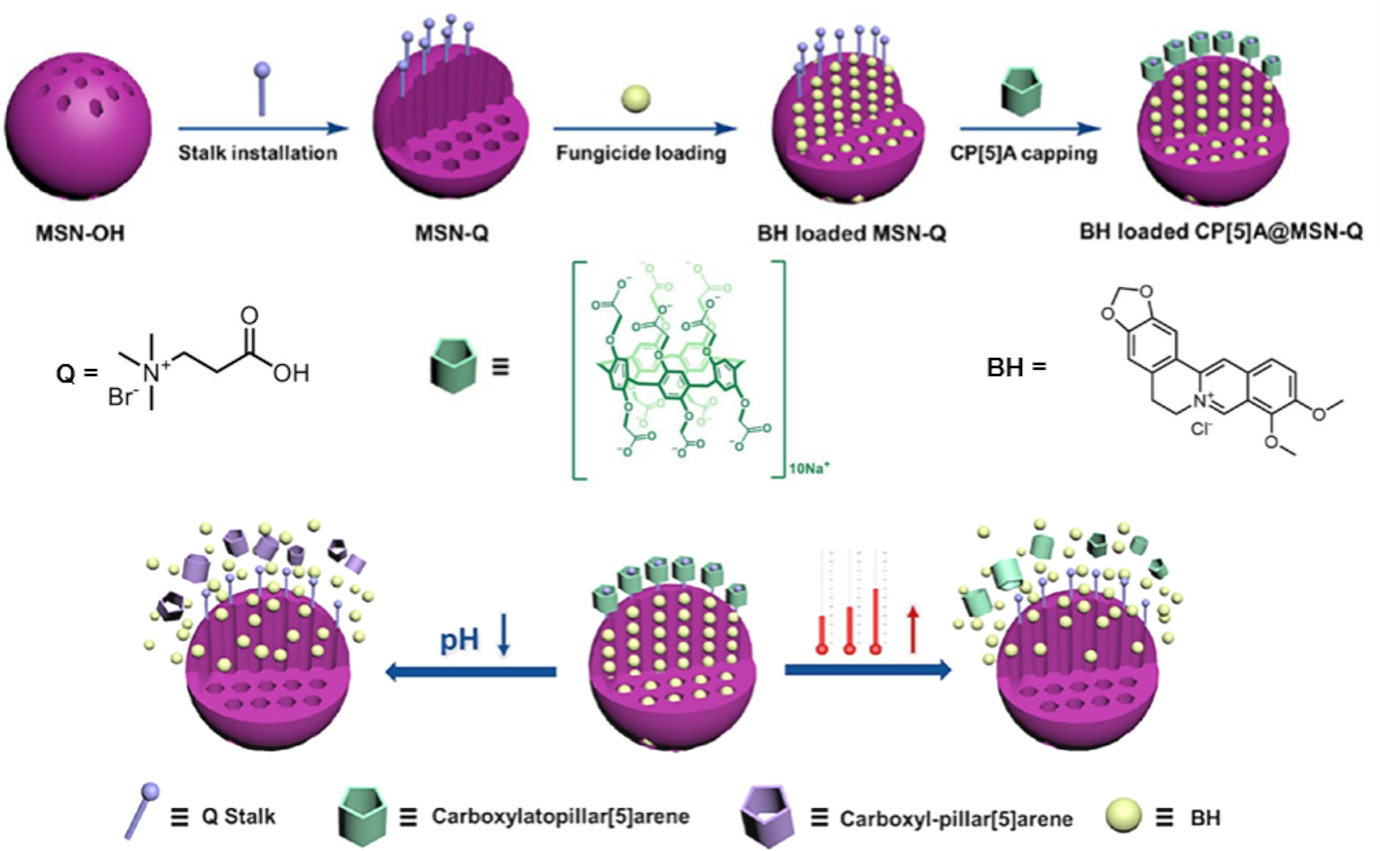
Illustration of the triggered release of BH from CP[5]A@MSNs-Q NPs in response to a drop in pH or a rise in temperature [133]. Figure 24 was adapted with permission from [133], Copyright 2021 American Chemical Society. This content is not subject to CC BY 4.0.

Intelligent cargo delivery systems have been utilized to develop pesticide delivery nanoplatforms, thanks to their controlled release characteristics. In 2021, Yang et al. engineered a supramolecular fungicide nanoplatform using quaternary ammonium salt (Q)-modified MSN (MSN-Q NPs) as nanocarriers to encapsulate berberine hydrochloride (BH) and carboxyl pillar[5]arene (CP5A) (Figure 24) [133]. CP5A acts as a nanogate and is anchored on the surface of MSN-P through host–guest interactions between the positively charged P groups and the electron-rich cavity of CP5A, forming CP5A@MSN-P NPs loaded with BH to combat Botrytis cinerea. CP5A imparted the nanoplatform with pH or elevating temperatures-stimuli-responsive release capabilities. The encapsulated BH, a natural plant-derived fungicide, served as an eco-friendly substitute for synthetic fungicides. They employed oxalic acid (OA) secreted by B. cinerea as a trigger to release BH on demand within the pathogen microenvironment. This study capitalized on the acidic microenvironment created by B. cinerea to construct a fungicide nanoplatform that releases fungicides on demand, distinguishing it from previous nanoplatforms that relied on exogenous stimuli. Results demonstrated that the nanoplatform effectively curbed mycelial growth and spore germination, offering a novel strategy for gray mold management.

**Figure 25:**
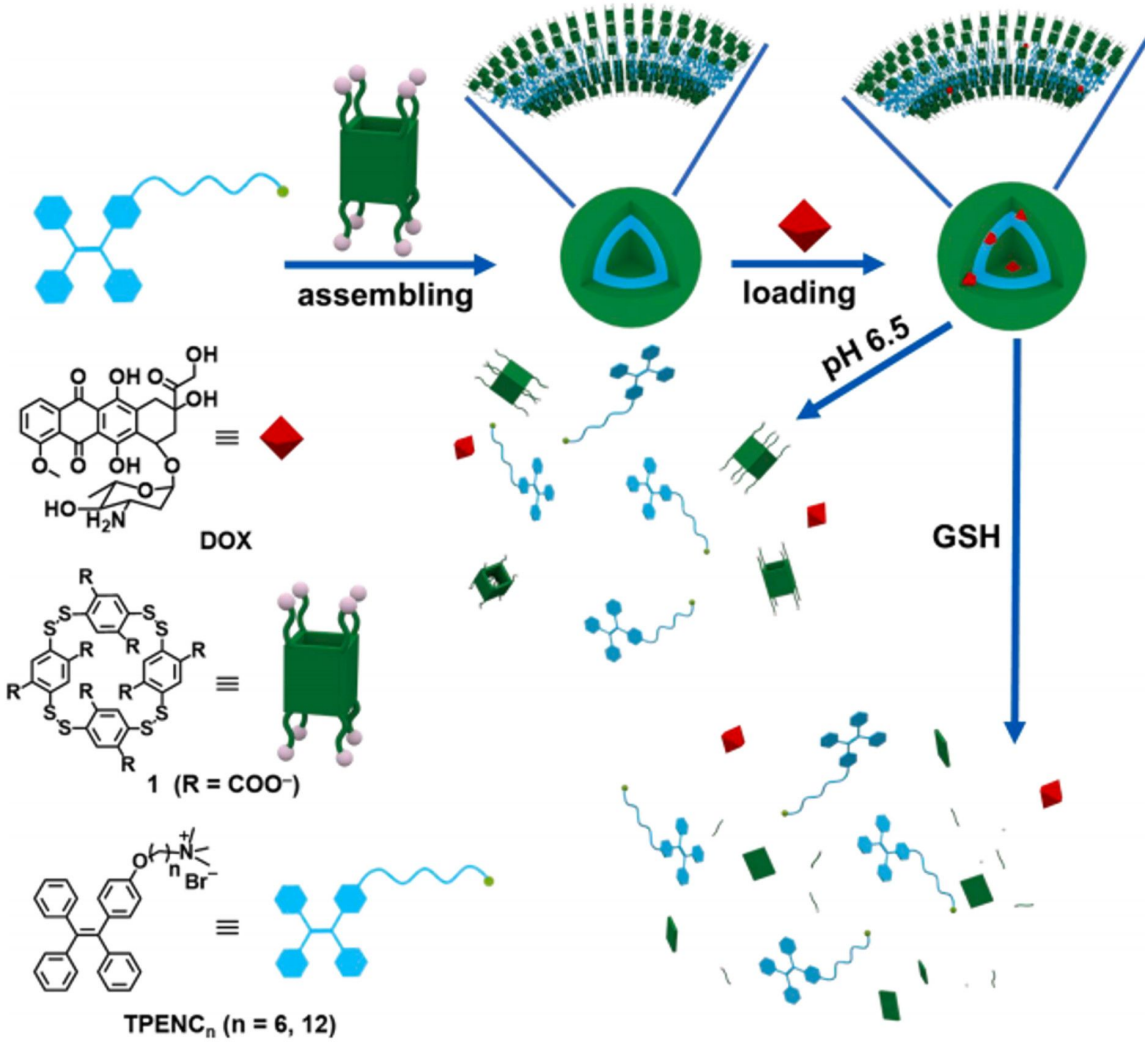
Illustration of the supramolecular amphiphiles TPENC_n_@1 (n = 6 and 12) self-assembling with disulfide bond-PA4 to form vesicles, which then load DOX and respond to acidic environments and overexpressed GSH for degradation [134]. Figure 25 was reprinted from [134], *Bioorganic & Medicinal Chemistry*, vol. 57, by Tang, M.; Liu, Y. H.; Xu, X. M.; Zhang, Y. M.; Liu, Y. “Dual-responsive drug release and fluorescence imaging based on disulfide-pillar [4] arene aggregate in cancer cells”, article no. 116649, Copyright (2022), with permission from Elsevier. This content is not subject to CC BY 4.0.

In 2022, Liu Yu and colleagues developed a pH and glutathione (GSH) dual-responsive supramolecular assembly by combining disulfide bond-containing pillar[4]arene (PA4) with tetraphenylethylene (TPE)-derived quaternary ammonium salts (TPENC_n_, *n* = 6, 12) (Figure 25) [134]. The system leverages the acidic pH and reductive microenvironment of cancer cells to trigger targeted drug release. PA4, featuring acid-labile carboxylate groups and reduction-sensitive disulfide bonds, forms amphiphilic nanoparticles with TPENC_n_ through electrostatic interactions, where the negatively charged PA4 pairs with the positively charged TPENC_n_. The hydrophilic segments consist of PA4’s carboxylate anions and TPENC_n_’s quaternary ammonium sites, while their aromatic backbones constitute the hydrophobic core. This assembly lowers the CAC of TPENC_n_, facilitating nanoparticle formation. Morphological studies confirmed well-structured nanoparticles optimized for cellular uptake. The dynamic non-covalent interactions enable stimulus-responsive behavior, allowing rapid drug release in acidic, high-GSH environments. Specifically, the TPENC6@PA4 nanoparticles demonstrated efficient DOX delivery, highlighting their potential as adaptive nanotherapeutics for cancer treatment.

## Conclusion

In summary, in recent years, supramolecular chemists have designed a series of easily synthesized aromatic macrocycles with electron-rich hydrophobic cavities. PAs and CAs, as intelligent hosts for drug-loading channels/holes, exhibit superior characteristics in supramolecular drug delivery systems. Most calix/pillar[*n*]arene hosts become water-soluble after modification with sulfonic acid, carboxyl, and phosphate groups. These calix/pillar[*n*]arene have good biocompatibility, ideal targeting ability, efficient operational capability, and precise response to various internal and external stimuli, and can be widely used as biomedicinal carriers. Researchers have connected functional groups that respond to specific stimuli to the aromatic macrocycle hosts. These specific functional groups endow supramolecular systems with rich responsiveness to light, pH, enzymes, and hypoxia-triggered, constructing different categories of supramolecular systems such as host–guest interactions, supramolecular self-assembly systems, and supramolecular nanovalves to achieve recognition, loading, and controlled entry and exit of drugs. It is worth mentioning that some of the constructed pH and enzyme-based controlled release systems can spontaneously generate stimulus responses in the tumor microenvironment without external human intervention to provide stimuli, thereby precisely releasing drugs, and have broad application prospects in cancer therapy.

Although there have been many reports on controlled release systems based on calix/pillar[*n*]arene in recent years, research in this field is still in its infancy, and it is challenging to conduct clinical trials for these systems. First, most stimulus-responsive nanosystems reported so far are based on SCA4 and CPA5−6. Compared to the extensive diversity of calix/pillar[*n*]arene, the currently developed types are just a tiny portion of the possible structures, greatly restricting the development of stimulus-responsive systems. Second, the known controlled release systems are usually limited to small molecules or ions, such as the anticancer drug DOX, the antibacterial MTZ, and ciprofloxacin. Due to the complexity and fragility of biomolecules, there are almost no reports on the encapsulation and release of larger biomolecules. Therefore, it is urgent to establish new stimulus-responsive controlled release systems that can contain large molecular drugs.

In view of the above, we hope that further improvement and overcoming can be achieved in future research. Different categories of aromatic macrocycles, such as tetralactam macrocycles and naphthotubes, can be tried to be modified with functional groups that respond to specific stimuli and used as hosts in release systems. Additionally, diverse stimulus-responsive systems derived from various group-modified aromatic macrocycles can be designed to align with the in vivo microenvironment, thereby facilitating the development of stimulus-responsive drug delivery systems. The photo-responsive systems based on CAs/PAs can be further optimized to improve their photo-responsive efficiency and stability. The applications of this system in more biomedical fields, such as targeted drug delivery and bioimaging, can be explored. The diversity of hypoxia-responsive systems can also be expanded by investigating CAs modified with different azobenzene structures for the treatment of tumor-related diseases. The drug release process is highly correlated with the disassembly of supramolecular nanoparticles. It can be anticipated that advanced drug loading and delivery efficiency combined with in situ monitoring of drug release will ultimately lead to effective chemotherapy and significantly inhibit tumor growth. In the future, the structure and properties of supramolecular amphiphiles can be further optimized to improve drug delivery efficiency and targeting ability. The applications of supramolecular amphiphiles in the treatment of other diseases can be explored, and the long-term safety and stability of supramolecular amphiphiles can be investigated. In the future, more biologically relevant molecules that have affinity for water-soluble CAs/PAs should be identified to achieve precise control over the size, shape, and stability of the assemblies. Other stimulus-responsive modes can be explored to expand the applications of these functional materials in other fields. Nevertheless, the clinical application of drug-controlled release systems based on aromatic macrocycles is still in its infancy. Extensive fundamental research and clinical trials are required to evaluate their safety, effectiveness, and practicality. Only through such rigorous assessment can these systems be more broadly implemented in clinical drug delivery, ultimately improving the precision and efficiency of drug administration and enhancing drug bioavailability. The development of supramolecular release systems new hope for improving drug targeting and bioavailability. Although there is still a need for innovation and progress in many aspects, we believe that with the in-depth research on stimulus-responsive host–guest chemistry, there is great potential in designing new therapies using release systems.

## Data Availability

Data sharing is not applicable as no new data was generated or analyzed in this study.
